# The Universally Conserved ATPase YchF Regulates Translation of Leaderless mRNA in Response to Stress Conditions

**DOI:** 10.3389/fmolb.2021.643696

**Published:** 2021-05-07

**Authors:** Victoria Landwehr, Martin Milanov, Larissa Angebauer, Jiang Hong, Gabriela Jüngert, Anna Hiersemenzel, Ariane Siebler, Fränk Schmit, Yavuz Öztürk, Stefan Dannenmaier, Friedel Drepper, Bettina Warscheid, Hans-Georg Koch

**Affiliations:** ^1^Institute for Biochemistry and Molecular Biology, Zentrum für Biochemie und Molekulare Medizin, Faculty of Medicine, Albert-Ludwigs-Universität Freiburg, Freiburg, Germany; ^2^Faculty of Biology, Albert-Ludwigs-Universität Freiburg, Freiburg, Germany; ^3^Spemann Graduate School of Biology and Medicine, Albert-Ludwigs-Universität Freiburg, Freiburg, Germany; ^4^Biochemistry and Functional Proteomics, Institute of Biology II, Faculty of Biology, Albert-Ludwigs-Universität Freiburg, Freiburg, Germany; ^5^Signalling Research Centers BIOSS and CIBSS, University Freiburg, Freiburg, Germany

**Keywords:** YchF/Ola1, protein synthesis, leaderless mRNA, translation control, stress, ribosomes

## Abstract

The universally conserved P-loop GTPases control diverse cellular processes, like signal transduction, ribosome assembly, cell motility, and intracellular transport and translation. YchF belongs to the Obg-family of P-loop GTPases and is one of the least characterized member of this family. It is unique because it preferentially hydrolyses ATP rather than GTP, but its physiological role is largely unknown. Studies in different organisms including humans suggest a possible role of YchF in regulating the cellular adaptation to stress conditions. In the current study, we explored the role of YchF in the model organism *Escherichia coli*. By western blot and promoter fusion experiments, we demonstrate that YchF levels decrease during stress conditions or when cells enter stationary phase. The decline in YchF levels trigger increased stress resistance and cells lacking YchF are resistant to multiple stress conditions, like oxidative stress, replication stress, or translational stress. By *in vivo* site directed cross-linking we demonstrate that YchF interacts with the translation initiation factor 3 (IF3) and with multiple ribosomal proteins at the surface of the small ribosomal subunit. The absence of YchF enhances the anti-association activity of IF3, stimulates the translation of leaderless mRNAs, and increases the resistance against the endoribonuclease MazF, which generates leaderless mRNAs during stress conditions. In summary, our data identify YchF as a stress-responsive regulator of leaderless mRNA translation.

## Introduction

Throughout their life, cells need to flexibly respond to changes in their environment. Consequently, sophisticated sensing and signal transduction systems have evolved in both eukaryotic and prokaryotic cells. These systems allow adjusting cell physiology in response to environmental and internal cues, and promote cell survival under stress conditions (Starosta et al., 2014). Stress responses primarily involve changes in gene expression and result in metabolic alterations, modification of enzymatic activities, and changes in protein homeostasis (de Nadal et al., 2011). The latter includes the adjustable synthesis of stress-response proteins, of which many are universally conserved and considered to constitute the minimal stress proteome.

Hallmarks of this adaptation are the selective induction of heat-shock proteins upon temperature shifts (Mogk et al., 2011) or antioxidant enzymes upon oxidative stress (Imlay, 2008). Although upregulation of stress response proteins is a common strategy for coping with stress conditions, other proteins are down-regulated and their absence seem to increase cellular fitness during stress. One example is the universally conserved ATPase YchF (Verstraeten et al., 2011; Balasingam et al., 2020). YchF belongs to the translation-factor-related (TRAFAC) class of P-loop GTPases, although it preferentially hydrolyses ATP rather than GTP, due to slight modifications in the active site (Koller-Eichhorn et al., 2007). The TRAFAC class of proteins comprises a functionally heterogeneous group of proteins, which include translation factors (Rodnina and Wintermeyer, 2016) and protein targeting factors (Steinberg et al., 2018), as well as proteins involved in ribosome assembly (Sato et al., 2005), cell cycle regulation (Foti et al., 2007), and stress response (Kuo et al., 2008).

In *Escherichia coli*, YchF is down-regulated when cells encounter oxidative stress and deleting YchF promotes cell survival under oxidative stress conditions (Wenk et al., 2012; Hannemann et al., 2016). A similar phenotype is observed in human cells, i.e., the knockdown of Ola1, the eukaryotic YchF homolog, results in increased resistance against oxidative stress (Zhang et al., 2009). Conversely, overproduction of YchF in *Arabidopsis thaliana* and *E. coli* inhibits the antioxidant response (Wenk et al., 2012; Cheung et al., 2013). This led to the hypothesis that YchF/Ola1 function as conserved negative regulators of oxidative stress response pathways (Zhang et al., 2009; Wenk et al., 2012; Cheung et al., 2013). However, the molecular mechanisms of this regulation are largely unknown. In *E. coli*, YchF is cross-linked to the catalase KatG and overproduction of YchF reduces catalase activity in cell extracts (Wenk et al., 2012). YchF also interacts with other antioxidant proteins like thioredoxin A (Hannemann et al., 2016) and therefore YchF could act by direct inhibition or trapping of antioxidant enzymes.

In addition, YchF/Ola1 are predicted to regulate protein synthesis. This is deduced from the observation that YchF interacts with ribosomes (Tomar et al., 2011) and that ribosomes stimulate its ATPase activity (Becker et al., 2012). Human Ola1 is suggested to regulate translation by converting the elongation initiation factor eIF2 into its GDP-bound state (Chen et al., 2015). Like the well-studied phosphorylation of eIF2 by eIF2 kinases (Dever et al., 1992), this would prevent binding of the initiator methionyl-tRNA to eIF2 and thus reduce translation initiation (Rodnina and Wintermeyer, 2009). On the other hand, the downregulation of Ola1 upon stress conditions (Sun et al., 2010) would increase translation initiation and attenuate the integrated stress response (ISR) (Chen et al., 2015; Pakos-Zebrucka et al., 2016). Consequently, the survival of stressed cells would be stimulated (Chen et al., 2015). Although this provides a tentative model for Ola1 function in humans, bacteria lack an eIF2 analog and the molecular mechanisms of translation initiation in bacteria and eukaryotes are significantly different (Rodnina et al., 2007; Rodnina and Wintermeyer, 2009).

A unifying concept for the evolutionary conservation of YchF/Ola1 proteins is thus still missing, which is why we further explored the function of YchF in the bacterial stress response.

## Materials and Methods

### Strains, Plasmids, and Growth Conditions

*Escherichia coli* BW25113 and BL21 were used as wild-type *E. coli* strains and were routinely grown on LB (Lysogeny broth) medium at 37°C. The *E. coli* strain JW1194 (Δ*ychF::km*) was provided by NBRP (NIG Japan) and grown on LB medium supplemented with 25 μg/ml kanamycin. The kanamycin cartridge of JW1194 was removed by FLP-mediated excision (Datsenko and Wanner, 2000), resulting in strain JW1194 (Km^S^). The conditional Ffh-depletion and FtsY-depletion strains Wam113 and IY28 have been described previously (Koch et al., 1999; Bürk et al., 2009). Strains carrying pBadYchF plasmids (Hannemann et al., 2016) were supplemented with 50 μg/ml ampicillin and strains carrying pSUP-BpaRS-6TRN or pEVOL (Chin et al., 2002; Ryu and Schultz, 2006) for *in vivo* cross-linking were supplemented with 35 μg/ml chloramphenicol. The pBad-YchF(N20pBpa)_Strep_ variant was constructed by PCR using the pBAD-YchF(N20pBpa)_His_ construct as template and the following primer YchFstrep-Fw: 5′-CCCGCAGTTCGAAAAATGAGATCCGGCTGCTAACAAAG CC-3′ and YchFstrep-Rv: 5′-TGGCTCCACGCCGAGACGTTG AAAAGGAAGTTCATCACATCG-3′. A pBadYchF-GFP-reporter plasmid was constructed via PCR cloning, fusing the C-terminus of YchF to GFP. Site-directed mutagenesis of pBadYchF for inserting TAG stop-codons was performed using inverse PCR and the Phusion High Fidelity PCR master mix New England Biolabs (NEB), Frankfurt, Germany. The *katG* gene was amplified from *E. coli* chromosomal DNA using the oligonucleotide primer KatG-fw (5′-ACATTGGGTCTCG-TATCATTACAGCAGGTCGAAACG-GTC-3′) and KatG-rev (5′-ACATTGGGTCTCAGCGC-CAT GAGCACGT-CAGACGAT-3′). The PCR product and the vector pASK17^+^ (IBA, Germany) were digested with *BsaI*, ligated and transformed into *E. coli* BL21. The P*_*y*__*chF*_*-GFP-reporter plasmid pGHS201 was obtained from the *E. coli* promoter collection [General Electric (GE)-Healthcare-Horizon, Lafayette, USA]. The plasmid pQE30*infC* was provided by T. Ueda (Tokyo University, Japan) (Shimizu and Ueda, 2010) and was co-expressed with the plasmid pRep4 (Qiagen, Germany) to control expression. For MazF-production, the plasmid pSA1 was used, which allows IPTG-dependent expression of mazF (Vesper et al., 2011). For monitoring translation of canonical and leaderless mRNAs, either the IPTG-inducible plasmids pMS2_512 and pMS2_53 (provided by Isabella Moll, University of Vienna, Austria) (Oron-Gottesman et al., 2016) or the arabinose-inducible plasmids pMG991 and pMG999 (provided by Frederica Briani, Università degli Studi di Milano, Italy) (Raneri et al., 2015) (Supplementary Material) were used. The coding sequences of the GFP-reporter of pMG991 and pMG999 were subcloned into the pCDFDuett vector (Novagen, Germany) by Gibson assembly using the following primer: pCDFDuet_frw: 5′-GGC AGC AGC CAT CAC CAT CAT C-3′; pCDFDuet_rev: 5′-CATGGTATATCTCCTTATTAAAGTT-AA ACAAAATTATTTC-3′; pMG_991_frw: 5′-CTTTAATAAGGA GATATACCATGACAGG-AGTAAAAATGGCTATCG-3′; pMG _991/999rev: 5′-TGATGGTGATGGCTGCTGCCCT-ATTTGT ATAGTTCATCCATGCC-3; pMG_999_frw: 5′-CTTTAATA AGGAGATATACCA-TGAGCACAAAAAAGAAACCATTAAC-3. The linear DNA of the vector was amplified from 10 ng, the isolated pCDFDuet using Q5 Polymerase Kit and using 10 μM of each primer. The obtained PCR product was DpnI-digested to remove the template. Using the same strategy, the fragments from pMG_991 and pMG_999 were amplified. The DNA fragments were assembled via Gibson assembly and the obtained plasmids pCDF-991 and pCDF-999 were verified by sequencing.

### Growth Analyses

*Escherichia coli* cells were grown overnight, diluted 1:100 in liquid LB medium and further incubated until they reached an OD_600_ of 0.5–0.8. These cells were then used for sensitivity assays by the following methods:

*(A) Spot assay*: The culture was adjusted to an OD_600_ of 0.5 and serially diluted. Twenty microliters of each dilution were spotted onto LB plates, supplemented with the corresponding stressor. After 20 h incubation at 37°C, the plates were analyzed.
*(B) Cell viability assay*: Cells were adjusted to an OD_600_ of 0.5 and diluted 1:10 in phosphate-buffered saline (PBS) before treatment with 10 mM H_2_O_2_ in PBS for 50 min at 25°C; control cells were treated with PBS. Subsequently, cells were harvested by centrifugation and washed with PBS and resuspended in 1 ml PBS. One hundred microliters of this cell culture was transferred to a 96-well plate and 100 μl of the BacTiter-Glo Microbial cell viability assay solution (Promega Corporation, Mannheim, Germany) was added. The luminescence of H_2_O_2_-treated wild-type cells was set to 100%.

For determining the *E. coli* cell length, overnight cultures were 1:100 diluted in fresh LB medium and grown to an OD_600_ of 0.5. The culture was then split into two cultures and after a further incubation for 1 h at 37°C, one culture was supplemented with 200 mM hydroxyurea, followed by a further incubation for 4 h at 37°C. The other culture served as control. Two-eight microliters of these cultures were then fixed on a microscope slide with 0.7% agarose and covered with a cover slide. Cells were then microscopically analyzed using an Olympus BX51 microscope with a numerical aperture of 1.4, an F-View charge-coupled device (CCD) camera and the cell^∗^F software (Olympus, Hamburg, Germany). Cell length of at least 400 individual cells was determined using the ImageJ-software.^1^ Data analyses were performed using Microsoft-Excel and GraphPad Prism 8.

### Competition Experiments Between Wild-Type and ΔychF Strains

Wild-type *E. coli* BW25113 and the Δ*ychF* (Km^R^) strain were inoculated from a single colony into 10 ml LB medium or LB + Km and incubated overnight at 37°C. From each culture, 5 × 10^8^ cells were used to inoculate 100 ml LB medium without antibiotics and incubated at 37°C with continuous shaking (180 rpm). At distinct time points, samples were removed and OD_600_ was determined. The samples were pelleted at 4,200 rpm for 10 min in a tabletop centrifuge, the cell pellet was resuspended in PBS-buffer and diluted to an OD_600_ of 0.1, corresponding to approximately 5 × 10^7^ cells/ml. By serial dilutions, the cell number was adjusted to 5 × 10^3^ cells/ml and 10 and 20 μl of this cell suspension were plated on LB-Agar plates ± Km. After incubation overnight at 37°C, the number of colonies (CFU, colony forming units) on the LB plate and the LB + Km plate were counted.

### SDS-Polyacrylamide Gel Electrophoresis (SDS-PAGE) and Western Blot Analyses

Samples were denatured at 95°C for 10 min. Samples for nonreducing SDS-PAGE were resuspended in DTT-free 4x Laemmli loading buffer (278 mM Tris-HCl, pH 6.8, 44.4% glycerol, 4.4% SDS,.02% bromophenol blue). Reducing loading buffer contained fresh DTT at a final concentration of 25 mM.

For western blot analyses, the proteins were blotted onto nitrocellulose membranes (GE Healthcare, Lafayette, United States). α-YchF antibodies were raised in rabbits against the peptide VNEDGFENNPYLDQC. KatG antibodies were obtained from Agrisera, Vännas, Sweden and the LexA antibody from Active Motif, La Hulpe, Belgium. Antibodies against YidC were raised against the purified protein (Koch et al., 2002). Antibodies against Pth were received from Gabriel Guarneros, Mexico City, Mexico; antibodies against the ribosomal proteins from Richard Brimacombe, Max Planck Institute for Molecular Genetics, Berlin, Germany; antibodies against IF3 from Isabella Moll, Vienna, Austria; and antibodies against MazF from Irina Marianovsky, Hebrew University of Jerusalem, Israel. A horseradish peroxidase-coupled secondary antibody was used for detection; blots were incubated for 1 min with homemade Enhanced Chemiluminescence (ECL) reagent and signals were detected by a CCD camera. Horseradish peroxidase (HRP)-coupled goat anti-rabbit or sheep anti-goat antibodies from Caltech Laboratories were used as secondary antibodies and homemade ECL reagent was used as detection substrate.

### Protein Purification

To purify N- or C-terminally His-tagged YchF or its pBpa-containing variants, BL21 was grown to an OD of 0.6–0.8 and induced with arabinose (pBad24-YchF 0.002%; pSUP 0.01%; pEVOL 0.02%) or 1 mM IPTG (pQE30*infC*+pRep4). After 3 h at 37°C, cells were harvested and resuspended in HKM buffer [25 mM HEPES (4-(2-hydroxyethyl)-1-piperazineethanesulfonic acid), pH 7.1, 200 mM KCl_2_, 10 mM MgCl_2_, 5% glycerol]. Complete protease inhibitor mixture (Roche, Germany) and phenylmethylsulfonylfluorid (PMSF), with final concentration 1 mM, were added. Cells were lysed three times by Emulsiflex C3 (Avestin) and cell debris was removed by centrifugation (30 min, 15,500 rpm, Thermo Scientific F21 rotor). The supernatant was mixed with HKM (+5 mM imidazole)-equilibrated Talon beads (Clontech) and incubated at 4°C for 1 h. The Talon beads were washed five times with 3 ml, 5 mM imidazole in HKM buffer and proteins were eluted with 200 mM imidazole in HKM buffer. Eluted proteins were buffer-exchanged using dialysis (Spectra/Por^TM^, MWCO 12–14 kDa) against HKMD buffer (25 mM HEPES, pH 7.1, 100 mM KCl_2,_ 7 mM MgCl_2,_ 10% glycerol) at 4°C before storage at –80°C.

KatG was purified via the N-terminal strep tag from *E. coli* BL21. The BL21 + pAsk17^+^KatG cells were cultured until OD_600_ of 1 and then induced with 200 μg/l anhydrotetracycline for 1 h. The cells were harvested by centrifugation at 5,000 rpm for 10 min at 4°C, resuspended in Buffer W (10 mM Tris-HCl pH 8; 150 mM NaCl, 1 mM EDTA) and lysed using a French pressure cell for three passages at 8,000 psi. To prevent degradation, 0.5 mM PMSF and 1× *Complete* protease inhibitor cocktail were added before French pressing. After centrifugation at 15,500 rpm, at 4°C for 30 min, the obtained S30 extract was loaded on a pre-equilibrated (2 ml buffer W) 1 ml strep-Tactin column (IBA, Germany). The column was washed by adding 5× 1 ml Buffer W. The protein was eluted with 3.5 ml buffer E (Buffer W + 2.5 mM desthiobiotin). Buffer was exchanged to 20 mM Tris-HCl pH 7.5 by PD-10 columns (GE Healthcare, Lafayette, United States). Purification of the Strep-tagged YchF followed the same protocol, with the exception that YchF production was induced by 0.002% arabinose.

High salt-treated ribosomes were purified via sucrose-gradient centrifugation as described previously (Bürk et al., 2009).

### Catalase Assay

KatG catalase activity was measured by monitoring its dismutation reaction 2 H_2_O_2_ + catalase → 2 H_2_O + O_2_. Purified KatG (4 μM final concentration) and when indicated, equimolar amounts of purified YchF, were incubated at 37°C for 20 min in 20 mM Tris-HCl, pH 7.5, and were injected into a sealed chamber to which after 1 min, 1 mM H_2_O_2_ was added. Oxygen release in the reaction chamber was monitored by a fiber optic oxygen meter (Fibox 3; PreSens GmbH, Regensburg, Germany) at 28°C and recorded by the OxyView 3.5.1 software (PreSens GmbH). The oxygen meter was calibrated with 1 ml, 1 mM H_2_O_2_ in 20 mM Tris-HCl, pH 7.5, with or without sodium dithionite before measurement.

### GFP-Reporter Assays

For monitoring the P*_*ychF*_*-GFP expression, cells were grown on M63 minimal media and at the indicated time points, 10^8^ cells were serially diluted in a black 96-well microplate with the transparent bottom (Greiner, Germany). Fluorescence was monitored at 510 nm after excitation at 380 nm using an Infinite M200 reader or the Spark plate reader (Tecan, Germany). The optical density of the culture was monitored in parallel by measuring optical density (OD) at 600 nm.

For monitoring the translation of canonical or leaderless mRNA, 10 × 10^8^ cells of a pre-culture were centrifuged at room temperature for 10 min at 5,000 rpm and resuspended in 500 μl M63 medium. Fifty microliter each of this sample were transferred into 10 separate tubes containing 5 ml M63 medium each. Cells were grown at 37°C and 180 rpm for 3 h (OD_600_ ∼0.4) and *gfp*-expression was induced by adding 1 mM IPTG or 0.2% arabinose. Two × 100 μl sample before and 2 h after induction of each tube were transferred into a black 96-well microplate with the transparent bottom (Greiner, Germany). Fluorescence was monitored at 535 nm after excitation at 485 nm using an Infinite M200 reader or the Spark plate reader (Tecan, Germany). The signal-to-background ratio (S/B) of each measurement was calculated by dividing the OD-normalized fluorescence signal after induction by the OD-normalized fluorescence signal before induction. For monitoring GFP fluorescence in the presence of Kasugamycin, cell cultures were incubated for 3 h, and then Kasugamycin was added, followed by 1 h incubation at 37°C. Expression of *gfp* was then induced and samples were processed as described above. For measuring GFP fluorescence of strains carrying pCDF-991 or pCDF-999, cells were grown on LB medium in the presence 0.002% arabinose and at OD_600_ = 0.4, the reporter plasmids were induced with 1 mM Isopropyl-β-D-thiogalactopyranosid (IPTG) and cells were grown for 90 min. Cells were harvested, washed in PBS, and resuspended in PBS. Samples were then processed as described above.

### *In vivo* Site-Directed Cross-Linking

*Escherichia coli* cells carrying pSUP-BpaRS-6TRN or pEVOL, and pBadYchF or its pBpa-containing derivatives, were grown overnight and used for the inoculation of 400 ml LB. After which, 0.5 mM pBpa in 1 M NaOH was added, and cells were grown at 37°C to an OD of 0.6–0.8 before they were induced with 0.002% arabinose. Cells were harvested after 3 h of growth and resuspended in 8 ml PBS buffer. Half of the sample was transferred into a six-well-microtiter plate and treated with UV-light [0.12 J/cm^2^) (UV Transilluminator Vilber Lourmat BLX-365 (Vilbert Lourmat, Eberhardszell, Germany)] for 20 min. The other half was protected against UV light. YchF was then purified from UV-exposed and control cells following the protocol described above.

### Ribosome Profiles

*Escherichia coli* cell cultures with a measurement of 1,000 ml were inoculated 1:100 from an overnight pre-culture and grown at 37°C. At an OD_600_ of 0.6 the culture was induced with arabinose (pEVOL: 0.02%; pSUP/pBad: 0.002%) or 1 mM IPTG. The cells were harvested at an OD_600_ of 1–1.2, and resuspended in CTF buffer (50 mM TEA, pH 7.5; 10 mM Mg-acetate, 5 mM K-acetate). Cells were lysed three times by Emulsiflex C3 (Avestin) and cell debris was removed by centrifugation (30 min, 15,500 rpm, Thermo Scientific F21 rotor). One hundred microliters of the supernatant was separated on a 20–50% sucrose density gradient (UltraPure Sucrose, Roth, Karlsruhe, Germany) in a swing-out rotor (JS-24.15, Beckman-Coulter) for 17 h at 29,000 rpm and 4°C. Samples were then fractionated and the RNA content per sample was simultaneously monitored via an UV detector at 256 nm. When indicated the fractionated samples were pooled according to the UV profile into the 30 S, 50 S, and 70 S fractions and stored at −80°C.

### Pulse Chase Experiments

Wild type and the Δ*ychF* strain were grown in M63 minimal medium (20 g/l glycerol; 13.6 g/l KH_2_PO_4_, 2 g/l (NH_4_)_2_SO_4_, 0.5 mg/l FeSO_4_, 200 mg/l MgSO_4_; 0.1 mM 18 amino acids, 0.025 mg/ml Thiamin) at 37°C after inoculation to a final OD_600_ of 0.2. At an OD_600_ of 0.3–0.4, MazF production was induced by the addition of 1 mM IPTG. After 1 h of induction, 1 × 10^8^ cells of each strain were harvested and resuspended in 2 ml M63 minimal medium containing 18 amino acids. Subsequently, 2 μl of ^35^S-labled methionine/cysteine labeling mix (Perkin Elmer, United States) were added and cells were further incubated with continuous shaking at 37°C. At indicated time points, 100 μl of cells were directly pipetted into 100 μl cold 10% trichloroacetic (TCA) solution. The proteins were precipitated for at least 30 min on ice and then centrifuged at 13,000 rpm and 4°C for 15 min. Each TCA pellet was resuspended in 25 μl loading dye and the sample was loaded on 5–15% gradient SDS-PAGE. Labeling was analyzed by phosphor-imaging of the dried gels by either the STORM 845 imager (GE Healthcare, Lafayette, United States) or the Typhoon TLA 7,000 imager (GE Healthcare) using the software *ImageQuant*.

### Mass Spectrometry

Samples for liquid chromatography-tandem mass spectrometry (LC-MS/MS) analyses were separated by SDS-PAGE. Following visualization of proteins with colloidal Coomassie Blue, gel lanes were cut into 13 slices and proteins were in-gel digested using trypsin as previously described (Peikert et al., 2017). Separation of peptides and MS analysis were performed using Ultimate 3,000 RSLCnano systems (Thermo Fisher Scientific, Dreieich, Germany), equipped with PepMap C18 precolumns (Thermo Scientific; length: 5 mm; inner diameter: 0.3 mm; loading flow rate: 30 μl/min) and Acclaim PepMap analytical columns (Thermo Scientific; length: 500 mm; inner diameter: 75 μm; particle size: 2 μm; packing density: 10 nm; flow rate: 0.25 μl/min) coupled online to an Orbitrap Elite mass spectrometer (Thermo Fisher Scientific, Bremen, Germany). Peptides were washed and concentrated on pre-columns and peptide separation was performed using a gradient of solvent A (4% DMSO; 0.1% FA) and solvent B (48% MeOH; 30% ACN; 4% DMSO; 0.1% FA). Separation and elution of peptides were performed using a multistep gradient of solvents A and B starting with 1% solvent B for 5 min followed by 1–65% B in 30 min, 65–95% B in 5 min, and 5 min at 95% B.

Mass spectrometry instruments were operated in data-dependent mode. Parameters were as follows: acquisition of full MS scans in the range of *m*/*z* 370–1,700; resolution of 120,000 at *m*/*z* 400; automatic gain control (AGC) of 1 × 10^6^ with a maximum allowed ion accumulation time (IT) of 200 ms; fragmentation of the 15 most abundant precursor ions with charge states ≥+2 (TOP15) by collision induced dissociation (CID); normalized collision energy of 35%; activation q of 0.25; activation time of 10 ms; AGC for MS/MS scans of 5 × 10^5^; IT of 150 ms; signal threshold of >2,500; dynamic exclusion time of 45 s.

### MS Data Analysis

Mass spectrometric raw data were processed using MaxQuant (version 1.5.5.1) (Cox and Mann, 2008) and its integrated search engine Andromeda (Cox et al., 2011). MS/MS data of YchF(N20pBpa) cross-linking experiments were searched against the *E. coli*-specific database from UniProt (release 2017_1). Database searches were performed with tryptic specificity and a maximum number of two missed cleavages. Mass tolerances were set to 4.5 ppm for precursor ions and 0.5 Da for fragment ions. Carbamidomethylation of cysteine residues was considered as fixed modification, and oxidation of methionine and N-terminal protein acetylation were set as variable modifications. The options “match between runs” and “iBAQ” were enabled. Proteins were identified with at least one unique peptide and a false discovery rate of 0.01 on both peptide and protein level. For the analysis of site-directed *in vivo* photo cross-linking experiments of YchF(N20pBpa), iBAQ intensities were used.

## Results

### YchF Is Involved in Regulating Multiple Stress Conditions

**FIGURE 1:**
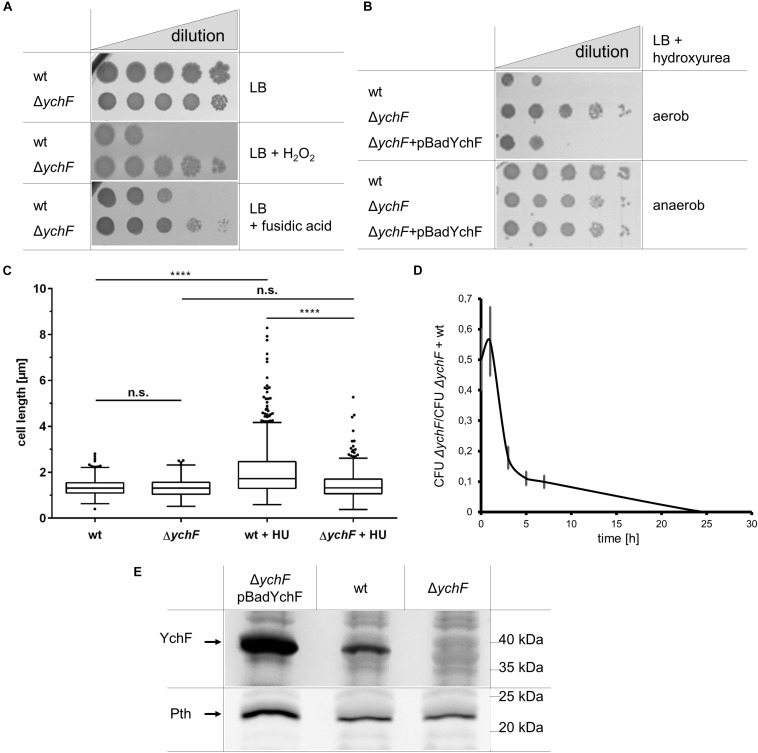
YchF is involved in regulating multiple stress responses in *E. coli*. **(A)** *E. coli* wild-type cells or the Δ*ychF* strain were inoculated in LB medium and grown to OD_600_ of ∼0.5 before serial dilution in LB medium. Of each dilution, 10 μl cell suspensions were spotted onto LB plates containing 2 mM H_2_O_2_ or 150 μg/ml fusidic acid when indicated. Cell growth was analyzed after overnight incubation at 37°C. **(B)** As in A, but cells were incubated on plates containing 7 mM hydroxyurea (HU) and 0.002% arabinose under aerobic and anaerobic conditions. Δ*ychF* + pBadYchF refers to the deletion strain carrying a plasmid-encoded *ychF* copy under the control of the arabinose promoter. **(C)** The indicated strains were grown on LB medium or LB medium with 7 mM mM HU and analyzed microscopically. *ImageJ* was used to determine the cell length of at least 400 individual cells. Values are shown as a box plot, in which the box reflects 75% of all measured values and the line inside of the box the mean value. The dots reflect values outside of the 95% confidence interval. The *p*-value was determined by a double *t*-test and (****) corresponds to *p* < 0.0001. n.s. not significant. **(D)** Equal numbers of wild-type and Δ*ychF*(Km^R^) cells were inoculated in LB medium as co-culture and at the indicated time points the number of colony forming units (CFU) on LB and LB-Km plates were determined. The mean values of *n* ≥ 3 experiments are shown and the error bars represent the standard error of the means (SEM). **(E)** 10^8^ cells of the indicated strains grown on LB medium in the presence of 0.002% arabinose were precipitated with 10% trichloroacetic acid (TCA) and the pellet was separated by SDS-PAGE, and after western transfer analyzed with antibodies against Pth and YchF. Representative images/gel blots of at least three independent experiments are displayed.

Previous data showed increased resistance of the Δ*ychF* strain against oxidative stress, induced by either H_2_O_2_ (Figure 1A and Supplementary Figure 1) or diamide, while YchF-overproducing cells were hypersensitive to oxidative stress (Wenk et al., 2012; Hannemann et al., 2016).

*In vivo*, YchF was shown to interact with catalase KatG by site-directed cross-linking/mass spectrometry, while cell extracts of YchF overproducing strains showed reduced catalase activity (Wenk et al., 2012; Hannemann et al., 2016). YchF was therefore suggested to function as a potential catalase inhibitor (Wenk et al., 2012). This was further analyzed by measuring KatG activity *in vitro* in the presence of purified wild-type YchF and YchF mutant variants. The KatG activity was unchanged in the presence of wild-type YchF (Supplementary Figure 2A). Immune detection revealed only a minor reduction of the *in vivo* steady-state amounts of KatG when YchF was overproduced (Supplementary Figure 2B). In *E. coli*, YchF is phosphorylated at serine residue 16 (Macek et al., 2008) and forms ATPase-deficient dimers upon oxidative stress (Hannemann et al., 2016). Therefore, KatG activity was also tested in the presence of the purified dimerization-deficient YchF variant YchF(C5/C35) (Hannemann et al., 2016), the phosphorylation-deficient YchF (S16A), the phospho-mimetic YchF (S16E), and the ATPase-deficient YchF(P11/N12) variants (Wenk et al., 2012), but no significant reduction of KatG activity was observed (Supplementary Figure 2C). Thus, the H_2_O_2_ hypersensitivity of the *ychF* overproducing strain and the increased H_2_O_2_ resistance of the Δ*ychF* strain are apparently not caused by changes in the KatG activity or its steady-state amounts.

Whether YchF has a more global impact on stress resistance in *E. coli* was tested by subjecting the Δ*ychF* strain to additional stress conditions. The Δ*ychF* strain exhibited also resistance against fusidic acid, an inhibitor of elongation factor G (EF-G; Rodnina and Wintermeyer, 2016; Figure 1A) and against hydroxyurea (HU), an inhibitor of ribonucleotide reductase (Spivak and Hasselbalch, 2011) (Figure 1B). HU resistance of the Δ*ychF* strain was not observed in the presence of an ectopic copy of *ychF*, demonstrating that resistance is linked to cellular YchF levels. However, it is important to note that the arabinose-induced production of YchF was not always completely uniform in all cells as monitored by a pBadYchF-GFP-reporter plasmid and therefore complementation assays were always controlled by western blotting. HU resistance was only observed under aerobic, but not under anaerobic conditions (Figure 1B). This is explained by the expression of the HU-resistant alternative ribonucleotide reductase NrdD under anaerobic conditions (Reichard, 1993; Fontecave et al., 2002). Inhibition of ribonucleotide reductase by HU prevents cell division and results in elongated *E. coli* cells (Renggli et al., 2013). Analyzing cell length of HU-treated wild-type and Δ*ychF* cells showed filamentous growth of the wild type, but normal cell morphology of the Δ*ychF* strain (Figure 1C).

While the absence of YchF confers a selective advantage when cells encounter stress conditions, the lack of YchF reduces fitness under non-stress conditions. This was determined by performing competition experiments between the wild type and the Δ*ychF* strain. In this experiment, a Km^R^ Δ*ychF* strain was used, while the other experiments were performed with a Km^S^ Δ*ychF* strain. An equal cell number of the wild type and the Δ*ychF* strain were used to inoculate an LB liquid culture and after different time points of co-culture, samples were spotted onto LB and LB + Km plates for determining the colony forming units (CFU). In these experiments, wild-type *E. coli* cells rapidly out-competed the Δ*ychF* cells (Figure 1D), indicating that the presence of YchF is important for competitive growth under non-stress conditions.

*YchF* in *E. coli* is co-transcribed with *pth*, which encodes the essential peptidyl-tRNA hydrolase (Cruz-Vera et al., 2002) and we therefore analyzed whether the deletion of *ychF* influenced the cellular Pth levels. However, there was no detectable difference in the Pth amounts between wild type and the Δ*ychF* strain (Figure 1E). Only in Δ*ychF* +pBadYchF cells was there an increase of Pth observed, which is in line with the reported stabilization of *pth* mRNA by the *ychF* transcript (Cruz-Vera et al., 2002). In summary, the absence of YchF results in an unusual gain-of-function phenotype that promotes cell survival under oxidative, translational, and replication stress.

### YchF Expression Is Growth Phase Dependent and Reduced Upon Stress Conditions

**FIGURE 2:**
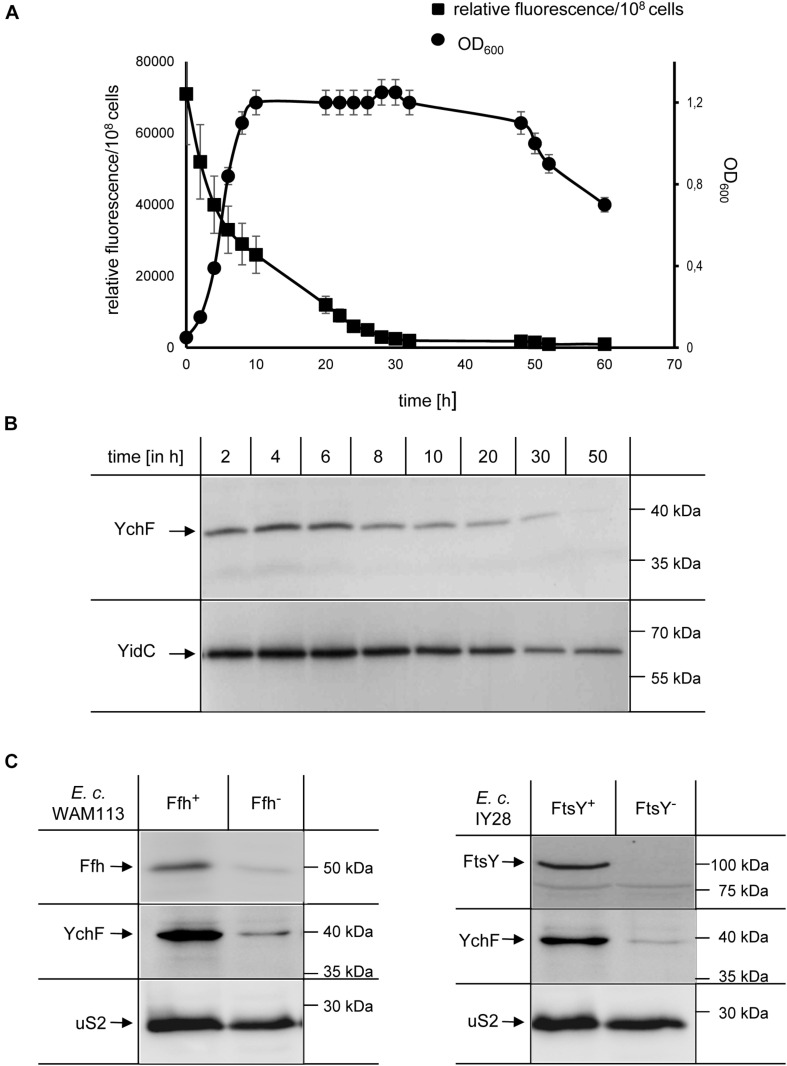
Growth-phase and stress-dependent downregulation of YchF. **(A)** Wild-type *E. coli* cells containing the green fluorescent protein (GFP) under control of the endogenous *ychF* promoter on a low-copy plasmid were grown on M63 minimal liquid medium, and the optical density (OD_600_) and GFP-fluorescence were monitored over time. The values shown represent the mean values of three independent experiments and the error bars indicate the standard error of the means (SEM). **(B)** At the indicated time points, 10^8^ cells of the culture in A were precipitated with 10% TCA and the pellet was separated by SDS-PAGE, and after western transfer analyzed with antibodies against YchF and YidC, which served as a control. Representative gel blots of at least three independent experiments are displayed and a quantification is shown in Supplementary Figure 3. **(C)** The conditional Ffh-depletion strain Wam113 and the conditional FtsY-depletion strain IY28 were grown on LB medium in the presence of 0.05% arabinose (Ffh^+^/FtsY^+^) or 0.5 % fructose (Ffh^–^ /FtsY^–^) for at least 3 h. A group of 5 × 10^8^ cells of the cultures were then precipitated with 10% TCA and the pellet was separated by SDS-PAGE, and after western transfer analyzed with antibodies against YchF, Ffh/FtsY, and against the ribosomal protein uS2.

Many stress-response pathways in *E. coli* are growth-phase dependent and therefore expression of *ychF* was monitored by fusing the *ychF* promoter to the GFP coding sequence and following GFP fluorescence in whole cells grown in M63 minimal media. The highest fluorescence was detected during the lag phase with a gradual decrease over time that reached background fluorescence in the mid-stationary growth phase (Figure 2A). The decrease of *ychF* expression was validated by immune detection, which showed that YchF levels decreased after approximately 6 h and were undetectable after approximately 50 h (Figure 2B and Supplementary Figure 3). As a control protein, levels of the inner membrane protein YidC were determined, which showed a much slower decrease and were detectable even after 50 h in significant amounts (Figure 2B and Supplementary Figure 3).

In *E. coli*, the steady-state levels of YchF are reduced when cells encounter oxidative stress and it was shown that the promoter region of *ychF* contains a typical binding motif for OxyR (Wenk et al., 2012), the main transcriptional regulator of the peroxide-induced stress response (Storz and Imlay, 1999). Whether the YchF levels are also reduced when *E. coli* encounters different stress conditions was analyzed in *E. coli* strains depleted for Ffh, the essential protein subunit of the bacterial signal recognition particle (SRP) or FtsY, the essential SRP receptor (Dalbey et al., 2017; Steinberg et al., 2018). Depletion of either Ffh or FtsY induces a multifaceted stress response that changes the cellular levels of many stress response proteins (Bernstein and Hyndman, 2001; Bürk et al., 2009; Wickstrom et al., 2011). When Ffh was depleted, a concomitant reduction of YchF was observed by western blotting and a similar YchF reduction was observed upon FtsY depletion (Figure 2C). In contrast, the levels of the ribosomal protein uS2 were not significantly changed. In summary, these data support a growth phase- and stress-dependent downregulation of YchF in *E. coli*. Furthermore, this indicates that stress-dependent downregulation is a conserved feature of the YchF/Ola1 protein family (Sun et al., 2010; Wenk et al., 2012; Chen et al., 2015).

### YchF Does Not Influence Steady-State Amounts of Ribosomes or Their Assembly

**FIGURE 3:**
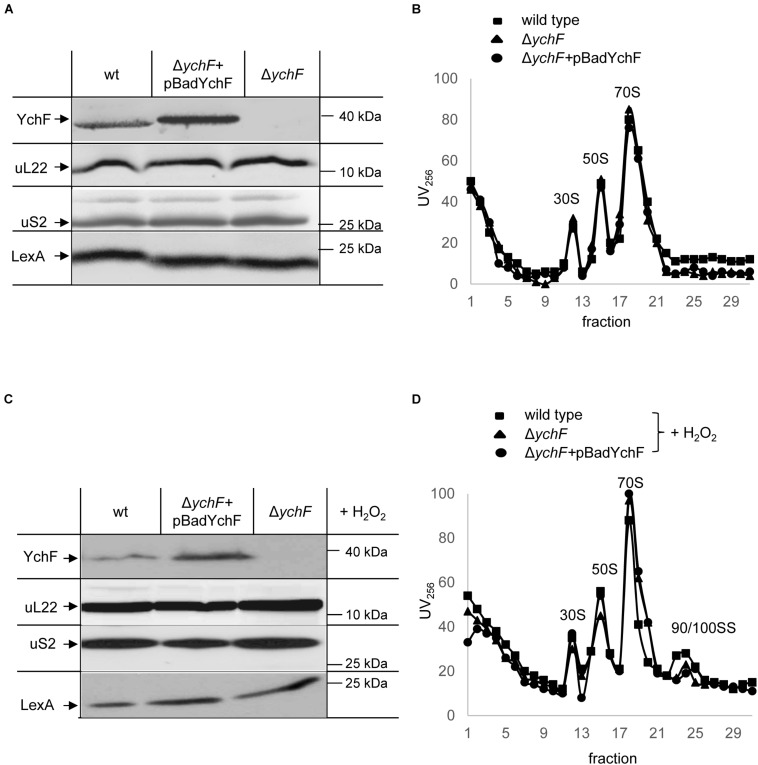
YchF does not influence the stability or assembly of ribosomes. The steady state amounts of ribosomes were analyzed in cell extracts of the indicated strains. Cells were grown on LB medium, harvested, and lysed by a French pressure cell. Expression of the plasmid-encoded *ychF* (pBadYchF) was induced with 0.002% arabinose. After determining the protein concentration in the crude cell extracts, equal amounts of protein were separated by SDS-PAGE and after western transfer, analyzed with antibodies against YchF, uL22, uS2, and LexA. **(B)** The crude cell extracts shown in panel **(A)** were subjected to sucrose-gradient density centrifugation (20–50% sucrose) and the individual ribosomal fraction was monitored at 256 nm for ribosomal RNA. Indicated are the 30 S, 50 S, 70 S ribosomal fractions. **(C)** As in panel **(A)**, but cells were treated with 20 mM H_2_O_2_ for 30 min at 37°C before harvesting. **(D)** As in B, the cell extracts of H_2_O_2_ treated cells were subjected to sucrose-gradient centrifugation and the 30 S, 50 S, 70 S, and 90/100 S ribosomal fractions are indicated. A quantification of three independent experiments is shown in Supplementary Figure 4.

Modulation of protein synthesis is a common response to cellular stress and decreasing protein synthesis by reducing the cellular ribosome concentration or by inhibiting ribosome activity is frequently observed (Bürk et al., 2009; Yoshida et al., 2009; Dai et al., 2016). In addition, the formation of dedicated ribosomes involved in synthesizing protective proteins has emerged as a new paradigm of the stress response (Moll and Engelberg-Kulka, 2012; Sauert et al., 2015). We therefore analyzed whether the steady-state amounts of ribosomes were influenced by the cellular YchF levels. Wild-type *E. coli* cells, and the Δ*ychF* strain and the Δ*ychF* strain expressing a plasmid-borne copy of *ychF*, were grown to mid-exponential phase and directly precipitated by TCA. After SDS-PAGE and western blotting, the membrane was analyzed with antibodies against ribosomal proteins uL22 of the 50 S ribosomal subunit and uS2 of the 30 S ribosomal subunit. There were no significant differences in the amount of uL22 or uS2 (Figure 3A), indicating that the three investigated strains contained comparable amounts of the large and the small ribosomal subunit. LexA, which is involved in DNA damage control (Baharoglu and Mazel, 2014), served as a loading control. The possible influence of YchF on ribosome assembly was further tested by obtaining ribosome profiles of the different strains after sucrose density centrifugation. Neither the deletion of *ychF* nor its overproduction significantly changed the occurrence of the 30 S, 50 S, and 70 S ribosomal fractions (Figure 3B and Supplementary Figure 4).

When cells were subjected to oxidative stress induced by H_2_O_2_, there was also no detectable difference in the amounts of ribosomal proteins (Figure 3C). The ribosome profiles of H_2_O_2_-treated cells were also not influenced by the cellular YchF content (Figure 3D and Supplementary Figure 4). Upon H_2_O_2_ treatment, an additional small peak was detected in all three strains (Figure 3D), which likely corresponds to the 90 S/100 S ribosomal fraction. This fraction is assigned to inactive 70 S dimers, which are transiently formed upon exposure to stress conditions (Bürk et al., 2009; Starosta et al., 2014). In conclusion, although YchF has been shown to interact with ribosomes (Tomar et al., 2011; Becker et al., 2012) and belongs to the Obg family of GTPases, which include many ribosome assembly factors (Verstraeten et al., 2011), we found no evidence that YchF influences the assembly or steady-state amounts of ribosomes in *E. coli* under stress or non-stress conditions.

### YchF Preferentially Interacts With the Small Ribosomal Subunit

**FIGURE 4:**
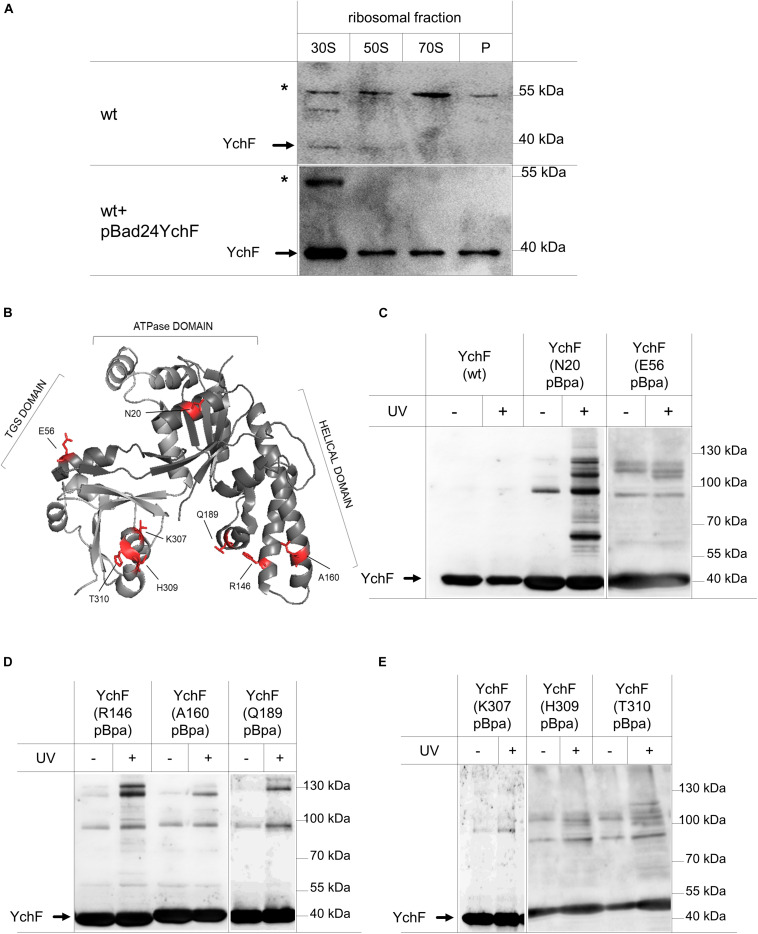
YchF preferentially binds to the 30 S ribosomal subunit. **(A)** The pooled 30 S, 50 S, and 70 S ribosomal fractions from wild-type cells or wild-type cells expressing a plasmid-borne copy of *ychF* (pBadYchF, induced with 0.002% arabinose) as shown in Figure 3, were analyzed by immune-detection with α-YchF antibodies. P correspond to the polysomal fractions and * indicates an unspecific band that is detected by the α-YchF antibody. Representative blots of several independent experiments are shown. **(B)** Crystal structure of *Haemophilus influenzae* YchF (pdb: 1JAL). The positions where para-benzoyl-L-phenylalanine (pBpa) was incorporated for *in vivo* site-directed cross-linking using the *E. coli* numbering, are indicated in red. **(C–E)** Cells expressing either wild-type (wt) YchF or YchF with pBpa incorporated at the indicated position were induced with 0.002% arabinose and UV exposed when indicated or kept in the dark. Cells were lysed and fractionated, and YchF and its potential cross-link partners were enriched by a single metal-affinity chromatography step. After SDS-PAGE and western blotting, the membrane was analyzed with α-YchF antibodies. Representative blots of at least three independent experiments are shown.

Ribosome binding of YchF/Ola1 has been demonstrated in different species (Olinares et al., 2010; Tomar et al., 2011; Becker et al., 2012; Chen et al., 2015), but the exact binding site of YchF on the ribosome is still under debate. For further analyses, ribosomal fractions of wild-type cells (Figure 3B) were analyzed by immune detection with α-YchF antibodies. Endogenous YchF was weakly detectable only in the 30 S ribosomal fraction (Figure 4A). The low ribosome occupancy of YchF is in line with its cellular concentration of approximately 3 μM (Becker et al., 2012), which is more than 10-fold lower than the cellular ribosome concentration (Kudva et al., 2013). In ribosomes from cells expressing a plasmid-borne *ychF* copy (Figure 3B), YchF was more readily detectable and enriched in the 30 S ribosomal fraction (Figure 4A), but also detectable in the 50 S, 70 S, and polysomal fractions. The less specific ribosome interaction of the plasmid-encoded YchF could reflect the moderate overproduction of YchF (Figure 3A). In addition, the presence of the N-terminal His-tag in YchF could favor unspecific interactions with the negatively charged ribosomal surface, in particular at lower pH values.

For analyzing the ribosome interaction of YchF further and for obtaining a more detailed view on the interaction surface between ribosomes and YchF, we executed an *in vivo* site-directed cross-linking approach. Site-directed *in vivo* photo-cross-linking was performed with para-benzoyl-L-phenylalanine (pBpa), an UV-sensitive amino acid derivative (Ryu and Schultz, 2006). pBpa can be specifically incorporated at amber stop-codon positions in the presence of a plasmid-borne orthogonal amino-acyl-tRNA synthetase/tRNA_CUA_ pair. The insertion of pBpa at different surface-exposed positions of YchF (Figure 4B) did not interfere with the YchF–ribosome interaction. This was tested for the YchF(N20pBpa) variant, which contained pBpa at position 20, close to the ATP binding site (Figure 4B). Sucrose gradient centrifugation demonstrated that YchF(N20pBpa) was preferentially found in the 30 S fraction, and to a lower extent also in the 50 S and 70 S fractions (Supplementary Figure 5).

Cells producing different pBpa-containing variants of YchF were exposed to UV-light for inducing potential cross-links, and then YchF and its cross-linked partner proteins were subsequently enriched by metal affinity chromatography. Cells producing wild-type YchF without pBpa, and cells that were not UV exposed, served as controls. UV exposure of wild-type cells did not result in any additional bands (Figure 4C), while UV exposure of YchF(N20pBpa)-producing cells resulted in several UV-dependent cross-linking products (Figure 4C). The most prominent products migrated at approximately 60 kDa, and between 100 and 130 kDa, but also several minor UV-dependent bands were detectable. In comparison, the other pBpa-containing YchF variants revealed only a few UV-specific cross-linking products (Figures 4C–E), demonstrating that in particular the N-terminal ATPase-domain of YchF is involved in protein-protein contacts. In all pBpa-containing YchF variants, a UV-independent band below the 100 kDa marker band was detected (Figures 4C–E), which likely reflects the YchF dimer that was reported previously (Hannemann et al., 2016), but this was not analyzed further.

**FIGURE 5:**
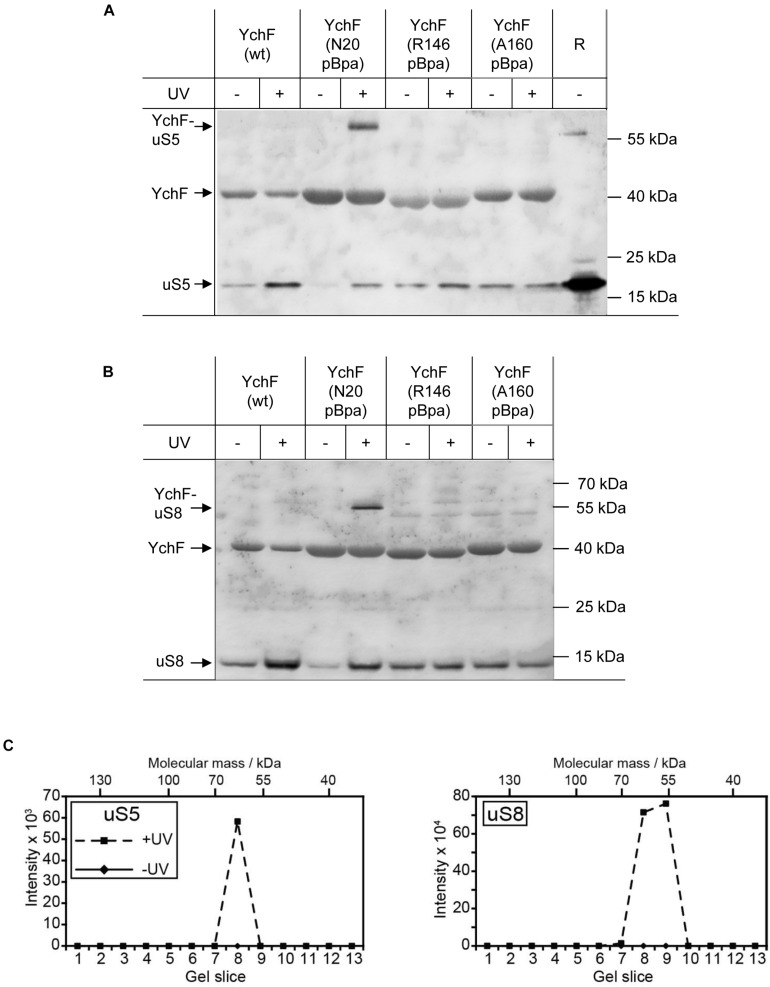
YchF binds to the ribosomal proteins uS5 and uS8. **(A,B)** The material described in Figure 4 was analyzed with antibodies against the ribosomal proteins uS5 and uS8. High-salt-treated purified ribosomes (R) were loaded as a control. Representative blots of at least three independent experiments are shown. **(C)** The gel lane of YchF(N20pBpa) was further analyzed by mass spectrometry after cutting the entire SDS-PAGE lane into slices, which were subjected to in-gel trypsin digestion. Detected peptide intensities divided by the number of possible peptides of a given protein from the UV-treated and the control sample (-UV) are shown.

Considering the preferred interaction of YchF with the 30 S ribosomal subunit, the enriched pBpa-containing variants were analyzed by immune detection using antibodies against proteins of the 30 S subunit that have been linked to the initiation and elongation cycle of the ribosome, like the ribosomal protein uS5. uS5 is located in the center of the 30S subunit and proposed to be involved in translational fidelity (Berk and Cate, 2007). Antibodies against the uS5 protein detected a UV-dependent band at approximately 60 kDa in YchF(N20pBpa), which is in line with the predicted mass of a cross-link between uS5 (17 kDa) and YchF (40 kDa) (Figure 5A). The antibodies also recognized free uS5 in the purified YchF material, suggesting that ribosomes—or at least uS5—co-purify with YchF on metal-affinity chromatography. Sucrose-gradient purified ribosomes served as a control for detecting free uS5 (Figure 5A, “R”). In the purified ribosomes, a weak band migrating below the predicted YchF-uS5 cross-linking product was also detectable. Furthermore, the uS5 antibodies also cross-reacted with purified YchF, which is probably caused by the high amount of purified YchF loaded onto the gel. The purified YchF sample from UV-treated cells and control cells were also analyzed by MS, which identified uS5 in the UV-treated but not in the UV-free control samples (Figure 5C, left panel). Only gel areas above 40 kDa were analyzed by MS, therefore the co-purifying uS5 was not detected in this analysis. YchF variants with pBpa inserted into the helical domain (residues R146 and A160, Figure 4B) were also analyzed with α-uS5 antibodies, but the α-uS5 antibodies detected no UV-dependent bands (Figure 5A), further demonstrating that it is, in particular, the N-terminus of YchF that interacts with the ribosome.

uS5 is located close to uS8, which is one of the primary 16S rRNA binding proteins (Yano and Yura, 1989; Jagannathan and Culver, 2003). Antibodies against uS8 detected a UV-dependent band at approximately 55 kDa for YchF(N20pBpa), which was not detectable in wild-type YchF or the YchF(R146pBpa)/YchF(A160pBpa) variants (Figure 5B). As also the uS8 antibodies cross-reacted with purified YchF, the identity of the cross-linked product was confirmed by mass spectrometry (Figure 5C, right panel).

**FIGURE 6:**
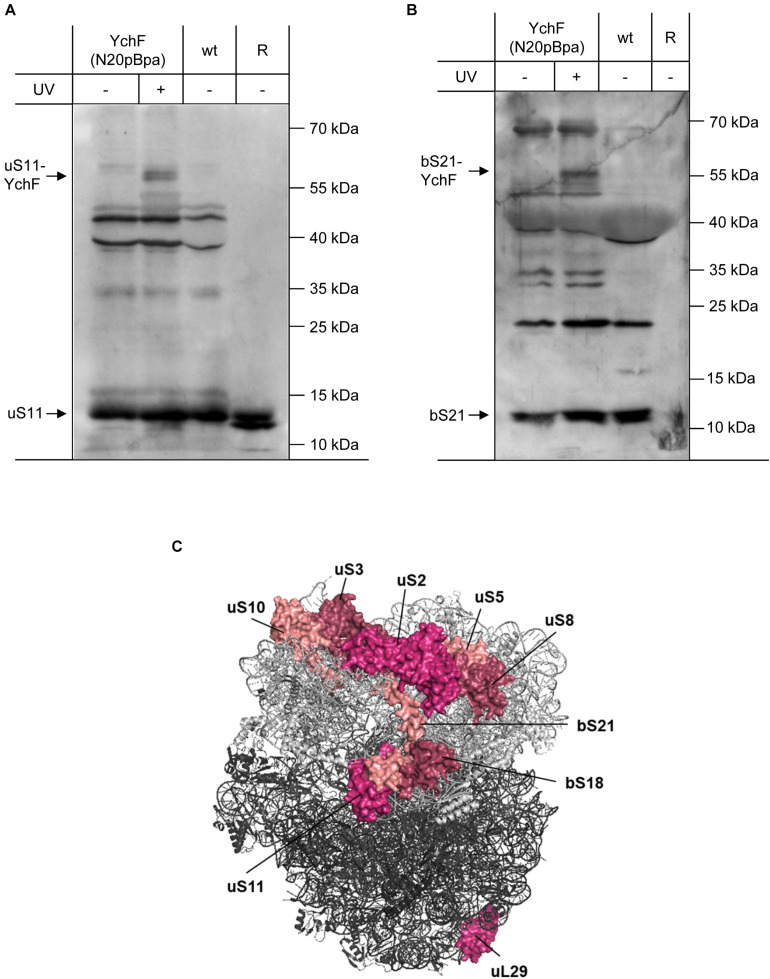
YchF interacts with the ribosomal proteins uS11 and bS21 **(A,B)** as described in Figure 5, but the enriched material was analyzed with antibodies against bS21 and uS11. As a control, purified ribosomes as in Figure 5 were also loaded. **(C)** Positions of ribosomal proteins that were found to cross-link to YchF. The 30 S subunit is displayed in light gray and the 50 S subunit in dark gray (PDB 5 mdz) (James et al., 2016); please note that the ribosomal proteins bS1 and bL7/12 are not visualized in this structure.

The ribosomal proteins uS5 and uS8 are extended toward the 30 S–50 S interface by the bacteria-specific ribosomal protein bS21 and the universally conserved protein uS11. uS11 forms part of the Shine-Dalgarno cleft in the 70 S ribosome and provides the contact site for initiation factor 3 (IF3). The cross-linking approach revealed UV-dependent cross-links of YchF(N20pBpa) to both uS11 and bS21 (Figures 6A,B). Further cross-links to bS1, uS2, uS3, uS10, and bS18 were also detected (Supplementary Figure 3), while no cross-links were observed to uS15 and uS20. In conclusion, the cross-linking data demonstrate that YchF binds via its N-terminal ATPase-domain preferentially to the central domain of the ribosomal 30 S subunit (Figure 6C).

The specificity of the interaction between YchF and the ribosome was further validated by using antibodies against proteins of the 50 S ribosomal subunit. Cross-links to the ribosomal stalk protein bL7/12 were observed (Supplementary Figures 3, 4). bL7/12 interacts with IF2 and this interaction is essential for rapid 30 S–50 S association. No cross-links to uL2, bL17, and uL18 were observed, but surprisingly, a cross-link to ribosomal protein uL29 was detected (Figure 6C and Supplementary Figures 6, 7). uL29 is located at the end of the ribosomal peptide tunnel and is part of an essential docking site for ribosome associated protein targeting factors and chaperones (Kramer et al., 2009; Denks et al., 2017; Knupffer et al., 2019). uL29 is located far away from the other identified ribosomal proteins and we therefore analyzed whether this interaction was caused by the presence of the N-terminal His-tag. However, when whole cells expressing a Strep-tagged version of YchF(N20pBpa) were analyzed, the cross-links to uL29 and uS11 were also detectable, indicating that the YchF–uL29 interaction is not the result of an unspecific interaction via the His-tag (Supplementary Figure 8). A possible explanation for the YchF-uL29 interaction is that YchF also interacts with polysomes (Figure 4A), which show a highly variable organization in electron tomography images (Brandt et al., 2009).

Additional contacts to ribosomal proteins of varying intensity were detected by mass spectrometry. However, we have been unable to confirm these contacts by immune detection, either because the available antibodies did not detect any UV-dependent band or because of the lack of suitable antibodies. Although our data show that YchF preferentially interacts with proteins of the 30 S ribosomal subunit, a particular docking site for YchF on the ribosome could not be identified. It is important to note that the cross-linking approach reports on proximity rather than on direct interaction because the spacer length of pBpa is approximately 10 Å (Wittelsberger et al., 2008). This could explain why several ribosomal proteins were cross-linked. In addition, YchF is suggested to bind to nucleic acids (Teplyakov et al., 2003), and the flexibility of ribosomal RNA (rRNA) on the ribosomal surface (Tian et al., 2010) could also rationalize the observation that YchF is not cross-linked to a single ribosomal protein. Finally, YchF has been shown to dimerize (Hannemann et al., 2016) and a YchF(N20pBpa) dimer would cover a larger surface on the ribosome and would also allow simultaneous cross-links to two distant ribosomal partner proteins.

### YchF Inhibits the Translation of Leaderless mRNAs by Modulating the Activity of IF3

**FIGURE 7:**
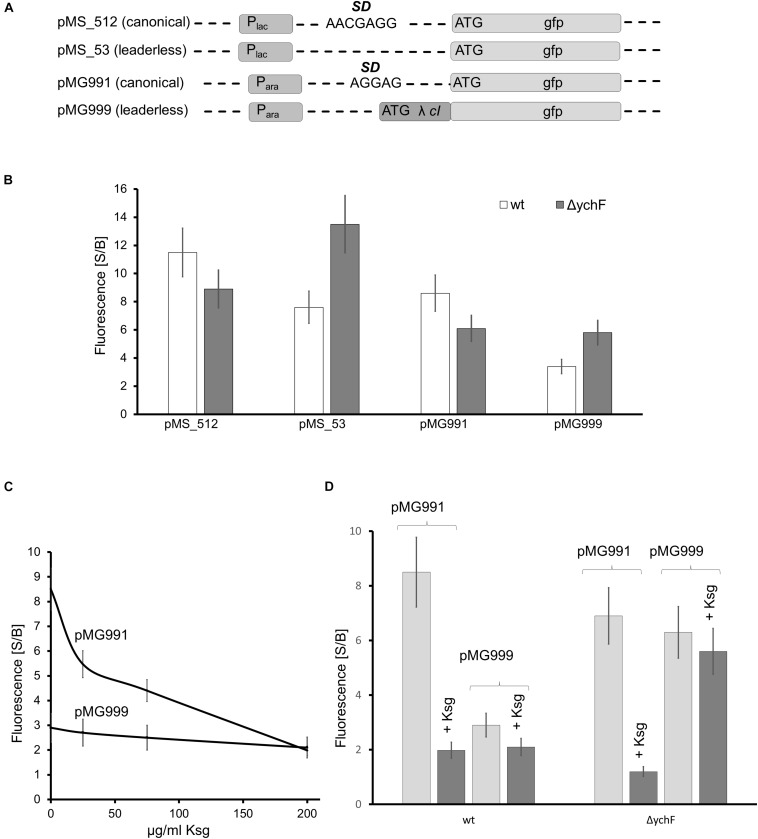
YchF is involved in the regulation of leaderless mRNA translation **(A)** Schematic view of the reporter constructs used in this study. P_lac_ refers to the lac promoter and P_ara_ to the arabinose promoter. SD, Shine-Dalgarno sequence; λ *cI*, repressor protein *cI* of phage λ; gfp, green fluorescent protein. **(B)** GFP fluorescence of wild-type and Δ*ychF* cells harboring the indicated plasmids. Cells were grown in M63 medium and GFP expression was induced by either 1 mM IPTG (pMS_512 and pMS_53) or 0.2% arabinose (pMG991 and pMG999) for 2 h before GFP fluorescence was measured. GFP fluorescence before (basal, B) and after induction (signal, S) was measured, normalized to the optical density of the culture, and is displayed as S/B ratio. The values of at least 12 independent experiments are shown and the bars represent the mean values and the error bar the SEM. **(C)** Wild-type cells harboring the indicated plasmids were grown as in panel **(B)**, but incubated with the indicated concentrations of kasugamycin (Ksg) for 1 h before induction. GFP fluorescence was measured as above. **(D)** As in panels **(B,C)**, but strains were incubated for 1 h with 200 μg Ksg/ml when indicated before induction. GFP fluorescence was measured as above.

The ability of YchF to interact with the ribosome and the observed stress resistance of the Δ*ychF* strain could indicate that YchF controls the selective translation of stress response proteins (Moll and Engelberg-Kulka, 2012). The human YchF homolog Ola1 was shown to inhibit translation initiation by binding to the initiation factor eIF2 (Chen et al., 2015). Bacteria lack an eIF2 analog and the molecular mechanisms of translation initiation are different compared with eukaryotes (Rodnina et al., 2007; Rodnina and Wintermeyer, 2009). However, under stress conditions, bacteria also translate mRNAs that lack the canonical Shine–Dalgarno (SD) sequence for ribosome binding (Moll and Engelberg-Kulka, 2012; Sauert et al., 2015). Translation initiation of these leaderless mRNAs (lmRNAs) possibly resembles translation initiation in eukaryotes (Udagawa et al., 2004; Beck and Moll, 2018); therefore, the influence of YchF on lmRNA translation was tested *in vivo* by using a translational GFP-reporter construct. Plasmid pMS_53 contains the GFP-coding sequence without a canonical SD sequence under the control of the lac promoter (Figure 7A and Supplementary Information). GFP-fluorescence of cells expressing this plasmid was compared to the GFP-fluorescence of cells expressing plasmid pMS**_**512, which contained a typical SD sequence upstream of *gfp*. In cells expressing pMS_512, i.e., in the presence of the SD, *in vivo* fluorescence in the Δ*ychF* strain was slightly lower than in the wild type (Figure 7B), but the opposite effect was observed for the leaderless construct in pMS_53. Here, the Δ*ychF* strain showed a higher fluorescence than the wild type (Figure 7B).

A possible inhibition of lmRNA translation by YchF was further validated by testing an independent reporter construct for lmRNA translation. Plasmid pMG999 contains the 5′-nucleotide sequence of the repressor protein *cI* from bacteriophage λ fused to GFP under the arabinose promoter (Figure 7A). The mRNA for *cI* is naturally leaderless and has been widely used in screens for inhibitors for leaderless translation (Raneri et al., 2015). Expression of pMG999 in Δ*ychF* cells resulted in higher fluorescence than in wild-type cells, while the expression of the control construct containing the SD (pMG991) showed lower fluorescence in the Δ*ychF* strain compared to the wild type. These data indicate that inhibitory effect of YchF on leaderless mRNA translation is independent of the genetic construct used for these assays.

Canonical translation is sensitive to the aminoglycoside antibiotic kasugamyin (Ksg), while the translation of lmRNA is Ksg resistant (Kaberdina et al., 2009). This allowed for a further control of the fluorescence assay by determining the fluorescence of pMG991/pMG999 expressing wild-type cells at increasing Ksg concentrations. GFP-fluorescence of cells expressing the SD-containing construct rapidly decreased in the presence of Ksg, but Ksg had no effect on the fluorescence of cells expressing the lmRNA construct (Figure 7C), which is in agreement with published data (Kaberdina et al., 2009; Raneri et al., 2015). A similar effect was observed in Δ*ychF* strains treated with Ksg: fluorescence of cells expressing the SD-containing construct was reduced, but fluorescence of cells expressing the lmRNA construct was unaffected by Ksg and higher than in the wild type (Figure 7D). These data further indicate that YchF inhibits the translation of lmRNAs in *E. coli*.

**FIGURE 8:**
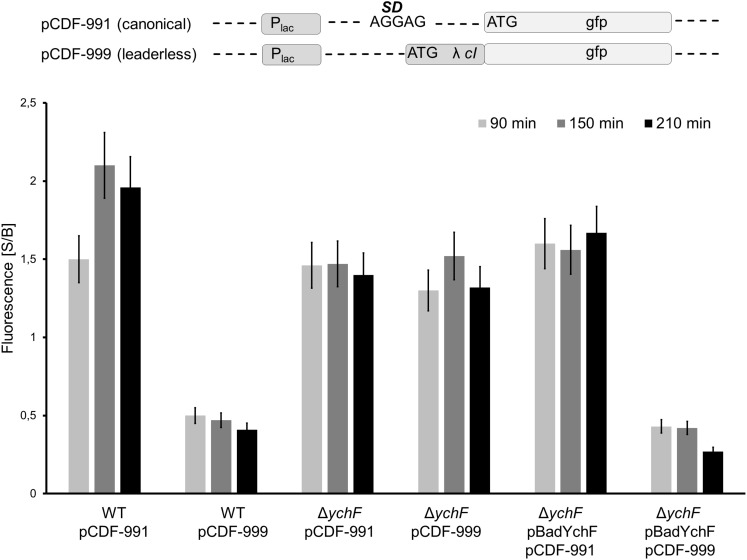
YchF reduces leaderless mRNA translation. The *gfp*-reporter sequence of plasmids pMG991 and pMG999 were cloned into the pCDFDuett vector, generating plasmids pCDF-991 (canonical translation) and pCDF-999 (lmRNA translation). The indicated strains were grown on LB-medium in the presence of 0.002% arabinose and at OD_600_ = 0.4, the expression of the reporter plasmids was induced with 1 mM IPTG and cells were grown at 37°C. At the indicated time points, cells were harvested, washed once in PBS and were then resuspended in PBS. Samples were analyzed as described in the legend in Figure 7. The mean values from at least three experiments are shown and the error bar reflects the SEM.

For analyzing the effect of YchF on lmRNA further, the GFP-reporter sequences of pMG999 and pMG991 were subcloned into the pCDFDuett vector, which is compatible with the pBadYchF plasmid. The plasmids pCDF-991 and pCDF-999 were then transformed into wild type, the Δ*ychF* strain and the Δ*ychF* strain containing pBadYchF and GFP fluorescence was determined. In support of the data shown above, lmRNA translation was impaired in wild-type cells, but comparable to canonical translation in Δ*ychF* cells (Figure 8). Importantly, in Δ*ychF* cells expressing a plasmid-borne *ychF* copy, lmRNA was significantly lower than canonical translation and corresponded to the values obtained for wild-type cells (Figure 8). Immune-detection confirmed the presence of YchF in the Δ*ychF* pBadYchF strain (Supplementary Figure 6). These data demonstrate that YchF inhibits the translation of lmRNAs.

**FIGURE 9:**
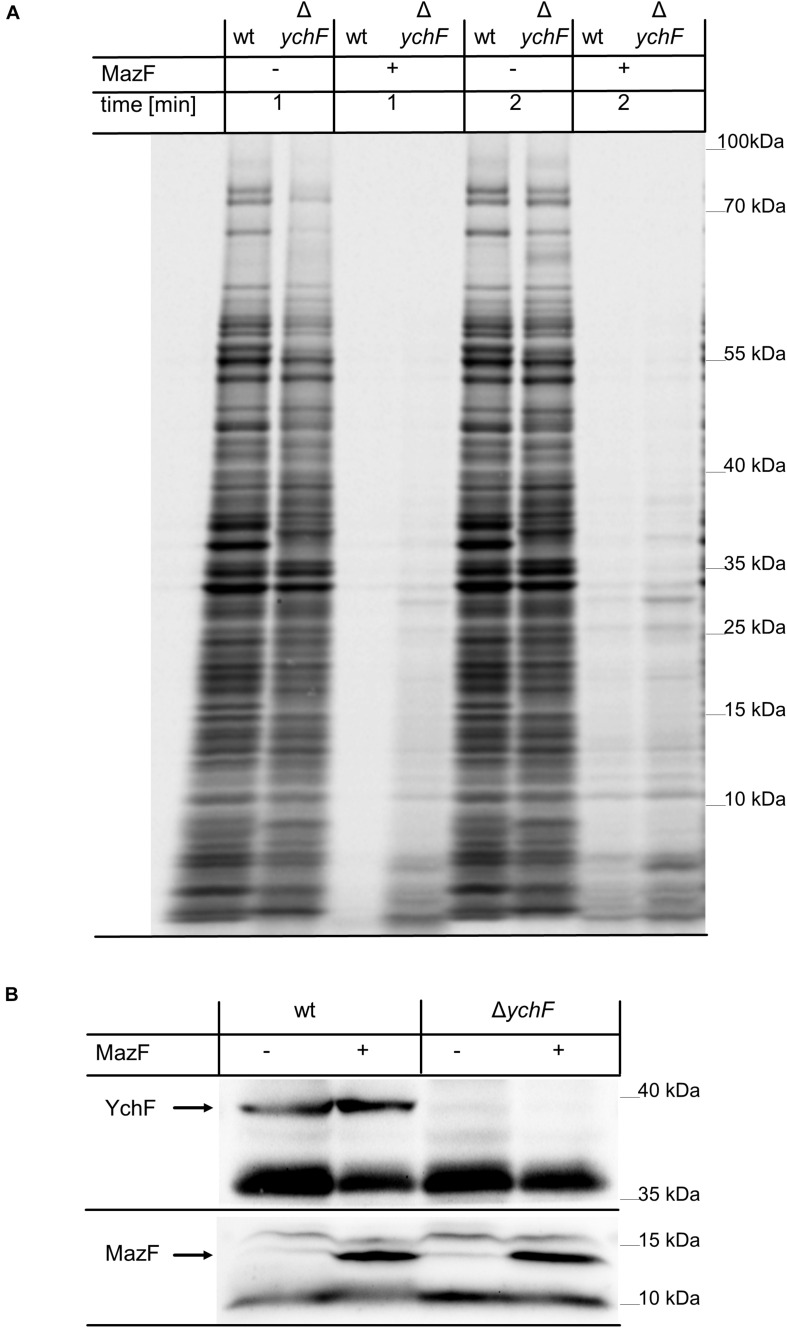
The absence of YchF increases resistance against MazF-dependent mRNA cleavage. **(A)** Plasmid-encoded *mazF* was expressed for one hour when indicated (+) in wild-type and Δ*ychF* cells, grown on M63 minimal medium. Subsequently, ^35^S-labeled cysteine and methionine were added and samples were taken after 1 and 2 min of labeling and directly TCA-precipitated. Samples were then separated by SDS PAGE and analyzed by phosphor-imaging. A representative image of at least three independent experiments is shown. **(B)** 10^8^ cells of the cultures before (0) or after induction (1) were precipitated with 10% TCA and the pellet was separated by SDS-PAGE and after western transfer analyzed with antibodies against YchF and His-tagged MazF.

*Escherichia coli* contains only a small number of lmRNAs during the exponential phase, but under stress conditions or when cells enter stationary phase, the number of lmRNAs increases (Beck and Moll, 2018). This is due to stress-induced production of the ribonuclease MazF, which degrades many mRNAs, but also cleaves some mRNAs close to the start-codon, and thus converts them into lmRNAs (Vesper et al., 2011; Sauert et al., 2016). Translation of these MazF-generated lmRNAs has been shown to be important for the survival of the bacterial population (Amitai et al., 2009). For analyzing the effect of YchF on MazF-induced mRNA cleavage and total protein synthesis, a pulse labeling experiment was performed. For simulating increased MazF levels, a plasmid-borne His-tagged MazF variant was expressed in both wild-type and Δ*ychF* cells for one hour before ^35^S-labeled methionine and cysteine were added to the exponentially growing cell culture. One or two minutes after labeling, samples were taken and directly precipitated by TCA. Newly synthesized and radioactively labeled proteins were then monitored by phosphor-imaging after SDS-PAGE. As a control, protein synthesis without MazF-production was analyzed in wild-type and Δ*ychF* cells. This revealed a comparable protein synthesis in wild-type and Δ*ychF* cells within the 1- and 2-min labeling period, although there were some variations in particular protein bands (Figure 9A). However, when MazF expression was induced, wild-type cells showed a drastically reduced protein synthesis, while Δ*ychF* cells were more resistant toward MazF-production (Figure 9A). This was not the result of variations in the MazF-levels, because western blotting using α-His antibodies revealed comparable amounts of MazF in the wild type and the Δ*ychF* strain (Figure 9B). The endogenous MazF levels were also not significantly influenced by the absence or presence of YchF (Supplementary Figure 9). In conclusion, YchF inhibits the translation of MazF-processed mRNAs, which is in line with an inhibition of lmRNA translation by YchF. However, MazF was also shown to regulate translation of several mRNAs by cleaving upstream of the SD sequence; for example, this was shown for the stress-induced transcription factor RpoS (Sauert et al., 2016). The increased protein synthesis in the MazF-expressing Δ*ychF* strain is therefore likely not exclusively the result of enhanced lmRNA translation.

**FIGURE 10:**
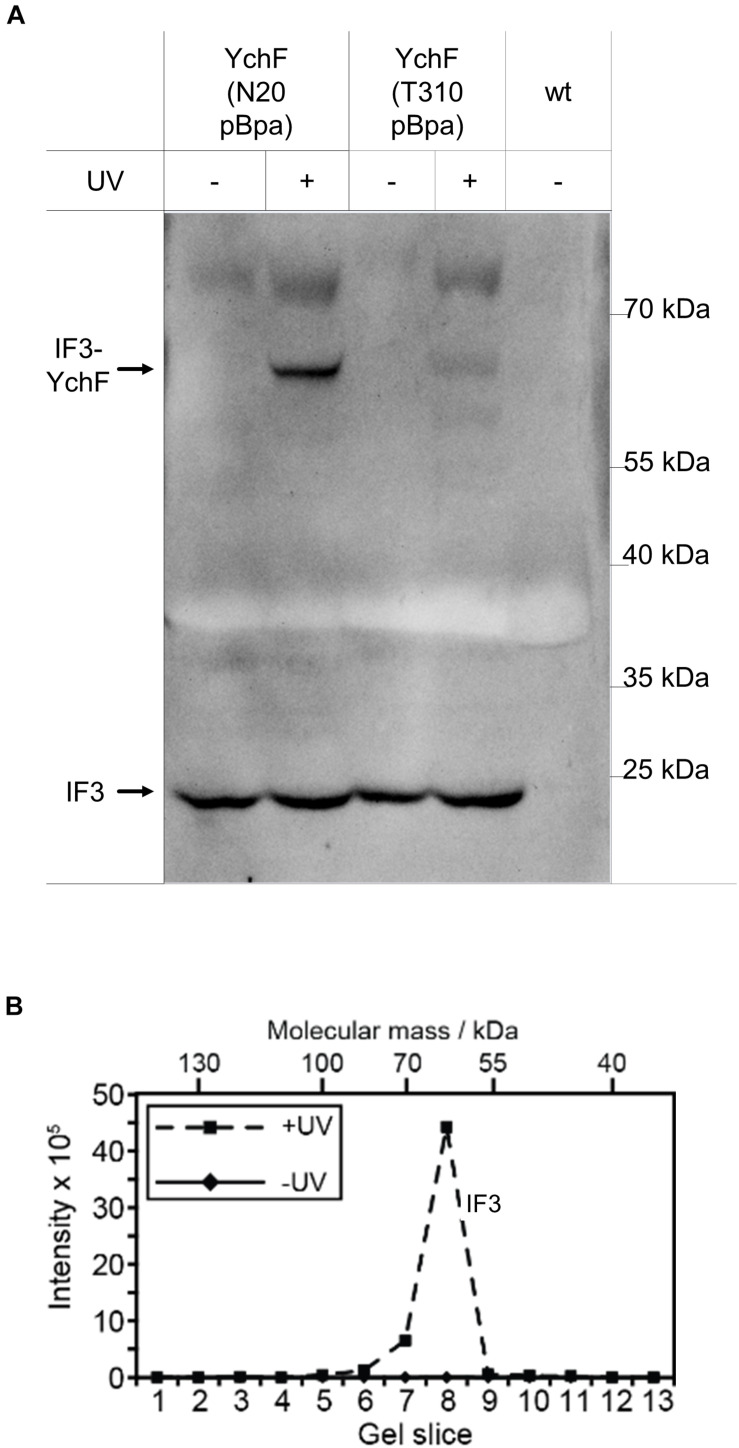
YchF interacts with initiation factor 3 (IF3). **(A)** The enriched material described in Figure 4 was analyzed with antibodies against initiation factor IF3. **(B)** The material in panel **(A)** was further analyzed by mass spectrometry after cutting the entire SDS-PAGE lane into slices, which were subjected to in-gel trypsin digestion. Detected peptide intensities divided by the number of possible peptides of a given protein from the UV-treated and the control sample (-UV) are shown.

Even with the combined data support of the inhibitory effect of YchF on lmRNA translation, details about the underlying mechanism are still unknown. In bacteria, the initiation factor 3 (IF3) discriminates against lmRNA translation and favors the formation of the canonical 30 S initiation complex (Tedin et al., 1997, 1999). The N-terminal domain of IF3 is located in the immediate vicinity to uS11 (Hussain et al., 2016), which was identified as cross-link partner of YchF (Figure 6A); therefore, a possible interaction between IF3 and YchF was tested by *in vivo* cross-linking. For YchF(N20pBpa), a strong YchF-IF3 cross-linking product was detected by western blotting (Figure 10A), while YchF(T310pBpa) showed only a weak cross-linking product (Figure 10A). The identity of the cross-linking product was further validated by mass spectrometry (Figure 10B). Independently of UV exposure, IF3 co-purified with both YchF variants (Figure 10A), further supporting an YchF-IF3 interaction. These results indicate that YchF binds to IF3 and that this interaction preferentially involves the N-terminal domain of YchF.

**FIGURE 11:**
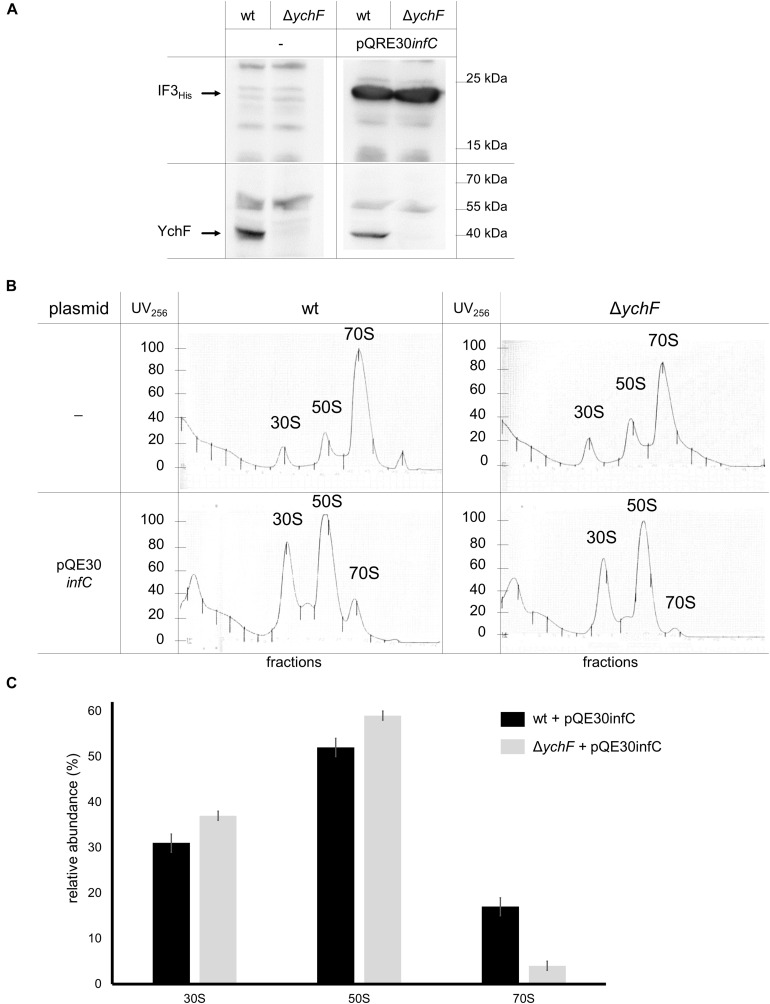
YchF counteracts the anti-associative effect of IF3 overexpression. **(A)** Wild-type and Δ*ychF* cells were co-transformed with plasmids pQE30*infC* and pREP4. After induction of *infC* expression, cells were harvested, lysed; and the supernatants after centrifugation were analyzed by immune-detection using α-His antibodies against His-tagged IF3 and α-YchF antibodies. **(B)** The supernatants of the cells in panel **(A)** were subjected to sucrose-gradient density centrifugation and ribosomal fractions were monitored photometrically at 256 nm. Representative traces of at least two independent experiments are shown. **(C)** Quantification of the ribosomal fractions of wild-type and Δ*ychF* strains expressing pQE30infC as shown in panel **(B)** (*n* = 2). The area below the curves was quantified using *ImageJ* and set to 100%. Areas corresponding to the 30 S, 50 S, and 70 S populations were extrapolated to the baseline, individually quantified and their relative abundance is shown.

Binding of IF3 to the 30 S subunit prevents the premature association of the 50 S subunit (Julián et al., 2011; Milón and Rodnina, 2012; Milón et al., 2012) and inhibits lmRNA translation (Yamamoto et al., 2016). In addition, IF3 also binds to the 50 S subunit, close to bL33, and this non-canonical binding is suggested to promote initiation on lmRNA on 70 S ribosomes (Yamamoto et al., 2016; Goyal et al., 2017); thus, one possibility is that YchF prevents initiation of lmRNA by regulating IF3 binding to either the 30 S or the 50 S subunit. This was further analyzed by expressing a plasmid-encoded His-tagged IF3 (*infC*_*His*_) in wild-type and Δ*ychF* cells. Monitoring the IF3 levels in cell extracts by western blotting using α-His antibodies revealed comparable amounts of IF3_His_ in wild-type and Δ*ychF* cell extracts (Figure 11A), while in plasmid-free cells the α-His antibody did not specifically recognize any band (Figure 11A). The ribosome pools in these cell extracts were then analyzed by sucrose-gradient centrifugation. Without IF3 overproduction, a typical distribution into 30 S, 50 S, and 70 S ribosomal pools were observed in wild-type and Δ*ychF* cells (Figure 11B). In contrast, wild-type cells overproducing IF3 showed increased amounts of 30 S and 50 S subunits, while the 70 S pool was reduced. This effect was even more pronounced in Δ*ychF* cells, which almost completely lacked the 70 S ribosome population (Figure 11B). Quantification of several ribosome fractionation experiments revealed only approximately 5% 70 S ribosomes in Δ*ychF* cells when IF3 was overproduced, versus approximately 20% in wild-type cells (Figure 11C). These data indicate that YchF can partially counteract the anti-associative effect of IF3 and support a direct interaction between YchF and IF3, as observed by the *in vivo* cross-linking data.

In summary, our data demonstrate that YchF is a ribosome-binding protein that specifically interacts with IF3 and inhibits the translation of lmRNAs. On the other hand, the stress-induced decrease of YchF or the decline of YchF when cells enter the stationary phase, promotes the translation of the concomitantly accumulating lmRNAs, which increases the stress tolerance and supports growth under non-favorable conditions.

## Discussion

Genomic studies have identified eight universally conserved GTPases, which cluster into four main GTPase families: the translation factor group, the FtsY/Ffh group, the Era group, and the Obg group (Verstraeten et al., 2011; Kint et al., 2014). YchF and its eukaryotic homolog Ola1 belong to the Obg group and are the least characterized GTPases (Balasingam et al., 2020), although there is accumulating evidence for their involvement in ribosome-associated processes (Becker et al., 2012; Samanfar et al., 2014; Chen et al., 2015; Ding et al., 2016) and stress response (Zhang et al., 2009; Cheung et al., 2010; Wenk et al., 2012; Chen et al., 2015; Hannemann et al., 2016).

A common response to stress conditions in eukaryotes and prokaryotes is the downregulation of ribosomal proteins and rRNA (Dennis and Nomura, 1974; Warner, 1999; Lemke et al., 2011; Starosta et al., 2014), which reduces protein synthesis and prevents the formation of misfolded or damaged proteins (Deuerling and Bukau, 2004; Tyedmers et al., 2010; Cherkasov et al., 2013; Brandman and Hegde, 2016); however, we did not find any indication that *E. coli* YchF had a significant influence on synthesis or assembly of ribosomes under stress conditions and therefore YchF does not appear to act as a regulator of ribosome production. This is also in line with a recent report showing that the absence of YchF does not influence the abundance of the 70 S, 50 S, and 30 S fractions in *E. coli* (Gibbs et al., 2020).

Ribosome binding of YchF/Ola1has been shown in eukaryotes and prokaryotes, but the exact binding site is still unknown. The available data indicate binding to the fully assembled 70 S/80 S ribosomes, but also binding to the 30 S/40 S and 50 S/60 S ribosomal subunits (Gradia et al., 2009; Tomar et al., 2011; Becker et al., 2012; Chen et al., 2015). In mammalian cells, Ola1 is preferentially located to the 40 S fraction and was shown to regulate the eukaryotic initiation factor 2 (eIF2)-mediated translational control. It has been suggested that Ola1 inhibits the formation of the ternary eIF2-GTP-tRNA^iMet^ complex (TC) by converting eIF2-GTP to eIF2-GDP (Chen et al., 2015). The reduced availability of the TC that usually delivers the tRNA^iMet^ to the 40 S ribosome (Myasnikov et al., 2009) would reduce translation initiation and potentially activate the ISR via selective translation of the transcription factor ATF4 (Vattem and Wek, 2004; Costa-Mattioli and Walter, 2020). ATF4, in turn, would then activate ISR (Wortel et al., 2017). The absence of Ola1, on the other hand, would attenuate ISR and promote cell survival under stress conditions. This provides an attractive model on how Ola1 could link translation initiation and stress response in eukaryotes; however, this model cannot be transferred to the bacterial YchF, because bacteria lack a homolog of eIF2. Instead, the tRNA^fMet^ is recruited by non-homologous GTP-bound IF2 to form a 30 S pre-initiation complex (Rodnina, 2018). Although the bacterial YchF might interact with IF2, YchF does not have a significant affinity for GTP (Tomar et al., 2011; Becker et al., 2012) and, thus, would be unable to convert GTP-IF2 into GDP-IF2. Even for the eukaryotic Ola1, there is conflicting evidence if Ola1 can indeed hydrolyze GTP (Koller-Eichhorn et al., 2007; Gradia et al., 2009; Chen et al., 2015).

Nevertheless, our data demonstrate that the endogenous and non-tagged YchF binds to the 30 S subunit in *E. coli*, which would be in line with a role during translation initiation, as predicted for Ola1. This would also agree with the general concept that translation is primarily controlled at the energy-consuming initiation state. However, it is important to note that plasmid-encoded and His-tagged YchF was also found in contact with the 50 S and 70 S ribosomes, as previously also observed by other groups (Tomar et al., 2011; Becker et al., 2012). The low abundance of endogenous YchF (Becker et al., 2012) possibly prevents its immune-detection in the 50 S/70 S ribosomal fraction. The binding pattern of YchF to the ribosome could be influenced by the presence of the His-tag, but YchF contacts to uL29 on the 50 S ribosomal subunit were also detected with a Strep-tagged YchF variant, suggesting that the N-terminal tag does not significantly influence ribosome binding. Alternatively, the YchF/ribosome ratio might influence the ribosomal contacts of YchF. This is also supported by mass-spectrometry analyses after *in vivo* cross-linking of His-tagged and plasmid-encoded YchF(N20pBpa), which identified many proteins of the 30 S and 50 S subunits as potential interaction partners. However, when the cross-linked material was analyzed by the less sensitive immune detection, available antibodies detected primarily proteins of the 30 S subunit. Intriguingly, most of the proteins determined as YchF-interacting proteins are located at the interface between the 30 S head and 30 S body (Hussain et al., 2016) and involve uS5, uS8, and uS11. uS11 also contacts IF3, which prevents the association of the 30 S initiation complex with the 50 S subunit, and is thus crucial for the fidelity of translation initiation. In support of the close proximity of YchF to uS11, our data also show that YchF contacts IF3.

It was recently shown that IF3 not only binds to the 30 S subunit, but also to the 50 S subunit in close proximity to uL33, which is located near the E-site of the ribosome (Goyal et al., 2017). The potential interaction between YchF and the 50 S-bound IF3 could also explain why YchF is cross-linked to proteins of the 30 S and 50 S subunits. Intriguingly, binding of IF3 to the 50 S subunit in 70 S ribosomes promotes translation initiation of leaderless mRNAs, while IF3 binding to the 30 S subunit prevents it (Yamamoto et al., 2016; Goyal et al., 2017). Leaderless mRNAs are present in all domains of life, but are more abundant in archaeal and bacterial genomes (Andreev et al., 2006; Christian and Spremulli, 2010; Beck and Moll, 2018). During stress conditions, the amount of lmRNAs increases due to the activity of the endoribonuclease MazF (Beck and Moll, 2018). MazF and its inhibitor MazE belong to the class II toxin-antitoxin systems (Cook et al., 2013). Upon stress-induced degradation of MazE, MazF is released and cleaves mRNAs both at the 5′-UTR and within the coding region (Sauert et al., 2016; Mets et al., 2017, 2019; Culviner and Laub, 2018). In addition, MazF also cleaves ribosomal RNA (Culviner and Laub, 2018). This causes a general reduction of protein synthesis and the selective translation of lmRNA, which enhances the cellular ability to cope with various stress conditions (Moll and Engelberg-Kulka, 2012). Intriguingly, among the predicted MazF-processed mRNA targets are *nrdAB* (ribonucleotide reductase) and *katG* (Sauert et al., 2016; Nigam et al., 2019), which are linked to the stress resistance observed for the Δ*ychF* strain; however, it is important to note that there is some controversy about the abundance of lmRNA produced by MazF (Culviner and Laub, 2018; Mets et al., 2019).

**FIGURE 12:**
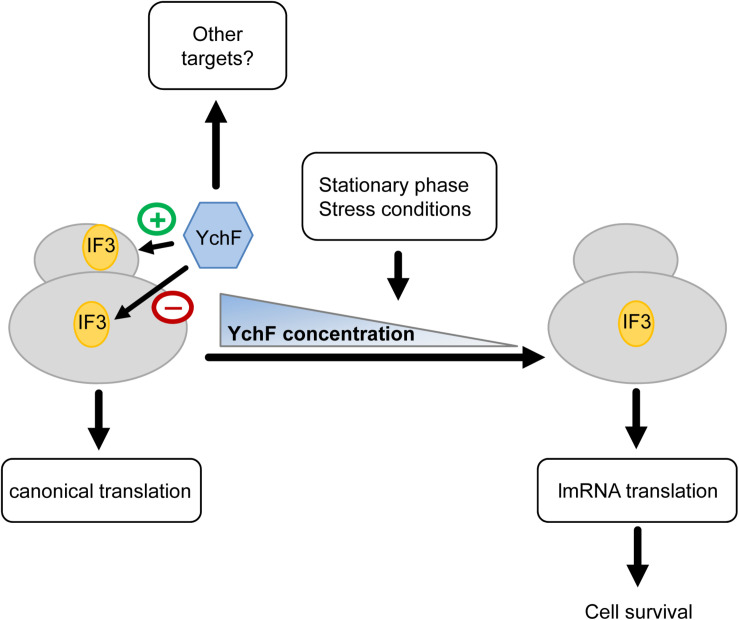
Putative model on YchF-dependent regulation of lmRNA translation. YchF promotes binding of IF3 to the 30 S ribosomal subunit and prevents binding of IF3 to the 50 S ribosomal subunit. This favors the translation of canonical mRNAs. When cells encounter stress or enter the stationary phase, the YchF levels gradually decrease, which allows binding of IF3 to the 50 S subunit, which in turn increases the translation of lmRNAs encoding stress response proteins and allows cell survival under stress conditions.

In summary, our data suggest that YchF is involved in regulating lmRNA translation and that it prevents/reduces lmRNA translation under non-stress conditions and during exponential phase (Figure 12). This is deduced from the observation that the absence of YchF: (1) increases lmRNA translation, (2) enhances the anti-associative activity of IF3 on the 30 S subunit, (3) increases the resistance to MazF, (4) increases stress resistance, but (5) reduces competitive fitness under non-stress conditions. The spatial and temporal regulations of IF3 binding to the ribosome is crucial for the preferential translation of these lmRNAs (Koripella et al., 2019), and IF3 binding to the 50 S subunit appears to be a critical step in this regulation (Goyal et al., 2017). Potentially, YchF could either enhance IF3 binding to the 30 S subunit or it could prevent IF3 binding to the 50 S subunit, but this requires further analyses. In both scenarios, YchF would sustain canonical translation under non-stress conditions, but when cells enter stationary phase or encounter stress conditions, the decreased YchF levels would favor translation of lmRNA and subsequent stress resistance. When cells encounter stress conditions, IF3 levels increase (Giuliodori et al., 2007), which might additionally enhance IF3 binding to the 50 S subunit and also lmRNA translation (Goyal et al., 2017). As lmRNAs are present in all domains of life, an involvement of YchF/Ola1 in lmRNA translation could also explain its universal conservation. This is also supported by data from yeast, which show that the YchF homolog Ola1 is enriched in heat-induced protein aggregates (Wallace et al., 2015). These aggregates contained many initiation factors that are required for the translation of canonical mRNAs but are dispensable for non-canonical initiation. Ola1 and heat-sensitive aggregation of initiation factors could serve as an alternative strategy in eukaryotes for promoting selective translation of stress-relevant mRNAs.

Nevertheless, it is important to note that several additional functions have been associated to the eukaryotic Ola1 and the connection to its role in lmRNA translation as postulated here is not directly obvious. This includes a possible role in centrosome regulation (Matsuzawa et al., 2014; Yoshino et al., 2018, 2019) or in the TGF-β/Smad signaling cascade, which controls cell growth and differentiation (Liu et al., 2020a,b). It is therefore possible that *E. coli* YchF does not only target ribosomes, but also—directly or indirectly—other players of the bacterial stress response. This could include RpoS, which is a major transcriptional regulator of the stress response (Schellhorn, 2020) and a potential MazF target (Sauert et al., 2016). YchF could also influence the synthesis of stress-signaling molecules, like (p)ppGpp (Haas et al., 2020; Steinchen et al., 2020) or chemical chaperones, like polyphosphate (Dahl et al., 2015), which are crucial determinants of the bacterial stress response. These possibilities are currently under investigation.

## Data Availability Statement

Raw data and result files have been deposited to the ProteomeXchange Consortium via the PRIDE repository (Perez-Riverol et al., 2019) with the dataset identifier PXD023192.

## Author Contributions

VL, MM, LA, JH, GJ, AH, YÖ, and H-GK contributed to the design of the study, acquisition, analysis, and interpretations of the data. AS, FS, SD, FD, and BW contributed to the acquisition, analysis, and interpretations of the data. All authors contributed to the article and approved the submitted version.

## Conflict of Interest

The authors declare that the research was conducted in the absence of any commercial or financial relationships that could be construed as a potential conflict of interest.

## Footnotes

1 https://imagej.nih.gov/ij/
