# Supplementary material for: The Universally Conserved ATPase YchF Regulates Translation of Leaderless mRNA in Response to Stress Conditions
Source: Front Mol Biosci. 2021 May 7;8:643696. doi: 10.3389/fmolb.2021.643696 (PMC8138138; doi:10.3389/fmolb.2021.643696)
Supplement: Supplementary file 1 [file Data_Sheet_1.PDF]

The universally conserved ATPase YchF regulates translation of leaderless mRNA in response to stress conditions

Victoria Landwehr[1,#], Martin Milanov[1,2,3,#],Larissa Angebauer[1,2], Jiang Hong[1,2], Gabriela Jüngert[1], Anna Hiersemenzel[1], Ariane Siebler[1], Fränk Schmit[1], Yavuz Öztürk[1], Stefan Dannenmaier[4], Friedel Drepper[4], Bettina Warscheid[3,4,5], Hans-Georg Koch[1,3]

[1]Institute for Biochemistry and Molecular Biology, Zentrum für Biochemie und Molekulare Medizin (ZMBZ), Faculty of Medicine, Albert-Ludwigs-Universität Freiburg, Freiburg, Germany

[2]Faculty of Biology, Albert-Ludwigs-Universität Freiburg, Freiburg, Germany

[3]Spemann Graduate School of Biology and Medicine, Albert-Ludwigs-Universität Freiburg, Freiburg, Germany

[4]Biochemistry and Functional Proteomics, Institute of Biology II, Faculty of Biology, Albert-Ludwigs-Universität Freiburg, Freiburg, Germany

[5]Signalling Research Centers BIOSS and CIBSS, University Freiburg, 79104 Freiburg, Germany

[#] Both authors contributed equally to this work

*** Correspondence:**
Hans-Georg Koch
Hans-Georg.Koch@biochemie.uni-freiburg.de

Keywords: YchF/Ola1, protein synthesis, leaderless mRNA, translation control, stress, ribosomes

Supplementary Material and Methods

Sequence information of plasmids pMS_53, pMS_512, pMG991,pMG999, pCDF-991 and pCDF-999.

The plasmids pMS_53 and pMS_512 were obtained from Isabella Moll, University Vienna, and the plasmids pMG991 and pMG999 from Frederica Briani, University Milano. A schematic view is displayed in Figure 7. Plasmids pCDF-991 and pCDF-999 were generated by inserting the GFP-reporter sequences of pMG991 and pMG999, respectively, into the pCDF-Duett vector backbone. The relevant difference between plasmids pCDF-991 and pCDF-999 and the original plasmids is that the GFP reporter sequence under the control of the lac promoter.

The plasmid pMG991 contains the first nine codons of *recA* (underlined) fused to *gfp* (highlighted in yellow) under the control of the arabinose promoter (P_{ara}, red). The Shine-Dalgarno sequence is indicated in red (SD). pMG999 contains the first 189 nucleotides of the naturally leaderless repressor protein *cI* of phage λ (underlined) fused to GFP under the arabinose-promoter.

The plasmid pMS_512 contains the GFP-coding sequence (highlighted in yellow) under the control of the lac promoter (red) and a strong Shine-Dalgarno sequence (SD). Plasmid pMS_53 contains the GFP-coding sequence under the lac promoter without SD.

pMG991

```
                         Para
AAAACACTTG ATACTGTATG AGCATACAGT ATAATTGTGG TACCCAACAG AACATATTGA CTATCCGGTA TTACCCGGCA
     SD                                           GFP
TGACAGGAGT AAAAATGGCT ATCGACGAAA ACAAACAGAA AGGTACC….
```

pMG999

```
                         Para
AAAACACTTG ATACTGTATG AGCATACAGT ATAATTGTAC CTGACGCTTT TTATCGCAAC TCTCTACTGT TTCTCCATAT
                                                λ cI
GAGCACAAAA AAGAAACCAT TAACACAAGA GCAGCTTGAG GACGCACGTC GCCTTAAAGC AATTTATGAA AAAAAGAAAA
ATGAACTTGG CTTATCCCAG GAATCTGTCG CAGACAAGAT GGGGATGGGG CAGTCAGGCG TTGGTGCTTT ATTTAATGGC
                                GFP
ATCAATGCAT TAAATGCTTA TAACGCCGGT ACC….
```

pMS_512

```
                     Plac
TTGACTTGTG AGCGGATAAC AATGATACTT AGATTCAGAA TTCTCGCCAG GGGTGCTCGG CATAAGCCGA AGATATCGGT
AGAGTTAATA TTGAGCAGAT CCCCCGGTGA AGGATTTAAC CGTGTTATCT CGTTGGAGAT ATTCATGGCG TATTTTGGAT
     SD                       GFP
CCTAACGAGG CGCAAAAAAT GGTGAGC….
```

pMS_53

```
                     Plac                                  GFP
TTGACTTGTG AGCGGATAAC AATGATACTT AGATTCCATG GTGAGC….
```


This is a provisional file, not the final typeset article

Legends to Supplemental figures

Figure S1. The absence of YchF allows cell survival under oxidative stress conditions. Wild type, Δ*ychF* and Δ*ychF* cells expressing an arabinose-inducible and plasmid-encoded *ychF*-copy were adjusted to OD_{600} of 0.5 and diluted 1:10 before treatment with 10 mM H_2O_2 in PBS for 50 min at 25 °C when indicated. After a washing step, 100 µl of the cell culture was transferred to a 96-well plate and 100 µl of the *BacTiter-Glo Microbial cell viability assay* solution was added and luminescence was recorded. The luminescence signal of wild type *E. coli* in the presence or absence of H_2O_2 was set to 100%. Shown are the mean values of at least three independent experiments and the error bars indicate the standard error of the means (s.e.m.). ** corresponds to $p< 0.01$.

Figure S2. YchF does not inhibit purified catalase KatG. (A) Purified KatG (4 µM) was incubated in a sealed reaction chamber and after 1 min 1mM H_2O_2 was added. O_2 release was monitored with a fibre optic oxygen meter. As a control, just buffer was incubated with H_2O_2 for monitoring spontaneous H_2O_2 dismutation. When indicated, equimolar amounts of YchF were added. Displayed are the means of at least three experiments. For clarity, the error bars are not displayed, but the s.e.m. was less than 5 µmol O_2/µg protein. (B) 10^8 cells of the indicated strains were precipitated with 10% TCA and the pellet was separated by SDS-PAGE and after western transfer the membrane was analysed with antibodies against KatG or LexA. (C) As in A, but O_2 release was monitored in the presence of equal amounts of different YchF variants. YchF(C5/C35) corresponds to a dimerization-deficient YchF variant, YchF(S16A) to a phosphorylation-deficient variant, YchF(S16E) to a phosphomimetic variant and YchF (P11/N12) to an ATPase-deficient YchF variant.

Figure S3. Growth-phase and stress-dependent down-regulation of YchF. *E. coli* wild type cells were grown on M63-medium and at the indicated time points, 10^8 cells were precipitated with 10% TCA and the pellet was separated by SDS-PAGE and after western transfer analyzed with antibodies against YchF and YidC, which served as a control. The chemiluminescence signals of three independent experiments were quantified using *ImageJ* and the signal at t= 2h was set to 100%. Displayed are the mean values and the error bar indicates the standard error of the mean (s.e.m.)

Figure S4. YchF does not influence ribosome assembly or steady state stability. (**A**) Cells were grown on LB medium, harvested and lysed by a French pressure cell. Expression of the plasmid-encoded *ychF* (pBadYchF) was induced with 0.002% arabinose. The crude cell extracts were subjected to sucrose-gradient density centrifugation (20-50% sucrose) and the individual ribosomal fraction were monitored at 256 nm for ribosomal RNA. The areas below the curves shown in Figure 3 were quantified using *ImageJ* and set to 100%. Areas corresponding to the 30S, 50S and 70S populations were extrapolated to the baseline, individually quantified and their relative abundance is shown. (**B**) As in A, but for ribosomal fractions derived from cells treated with 20 mM H_2O_2 for 30 min at 37°C before harvesting. Values represent the mean values of three independent experiments and the error bar indicates the standard error of the mean (s.e.m.).

Figure S5. The incorporation of pBpa into YchF at position N20 does not interfere with ribosome binding. Crude extracts of cells expressing pBad24YchF(N20pBpa), which encodes a YchF variant that was used for *in vivo* site-directed cross-linking using the amino acid derivative pBpa incorporated at position 20 of YchF were fractionated on sucrose gradients as described in the legend to Figure 3. After western blotting, fractions were analysed with α-YchF antibodies. * indicates a band that is un-specifically recognized by the antibody.

Figure S6. YchF interacts with ribosomal proteins bS1, uS2, uS3, uS10, bS18, bL7/12 and uL29 but not with uS15, uL2, bL17 and uL18. After *in vivo* cross-linking, samples were processed as described in the legend to Figure 5 and analysed with antibodies against the indicated ribosomal proteins. Wt corresponds to *E. coli* cells expressing wild type YchF, lacking pBpa and R to high-salt treated purified ribosomes.

Figure S7. Identification of YchF cross-linking to ribosomal proteins by mass spectrometry. After SDS-PAGE of the enriched material of YchF(N20pBpa) expressing cells before or after UV exposure, the entire gel lane was cut into slices and subjected to an in-gel trypsin digestion of the individual gel slices. Detected peptide intensities of each protein were divided by the number of expected peptides to correct for varying protein sizes and both the UV-treated (+ UV) and the control sample (-UV) are shown.

Figure S8. Ribosomal contacts are also observed for a Strep-tagged YchF(N20pBpa) variant. Wild type cells containing either pBadYchF$_{His}$(N20pBpa) or pBadYchF$_{Strep}$(N20pBpa) were grown on M63 medium in the presence of 0.5 mM pBpa and at an OD_{600} of 0.5 YchF production was induced by the addition of 0.01% arabinose. After 3 h of growth, cells were harvested, resuspended in PBS buffer and one half was exposed to UV-light for 30 min on ice, while the other half was kept in the dark. Cells were then TCA precipitated and directly loaded on a 5-15% SDS-PAGE. After western blotting, membranes were either incubated with antibodies against uL29 (upper panel) or uS11 (lower panel). Note, that in comparison to the other cross-linking experiments, the cross-linking products were not purified, but whole cells were analysed. This was necessary because the Strep-tagged YchF(N20pBpa) could not be purified, probably due to cleavage of the Strep-tag after cell lysis.

Figure S9. YchF inhibits leaderless mRNA translation. The *E. coli* cells analysed for canonical and lmRNA translation as described in Figure 8 were analysed for the YchF content (A) and for the MazF content (B) after 210 min of induction by using polyclonal antibodies against the native proteins. For preventing any cross-contamination, the blots in (A) and (B) show the strains in a different order and the samples in (A) were separated by empty lanes and a molecular weight marker (MW).

This is a provisional file, not the final typeset article

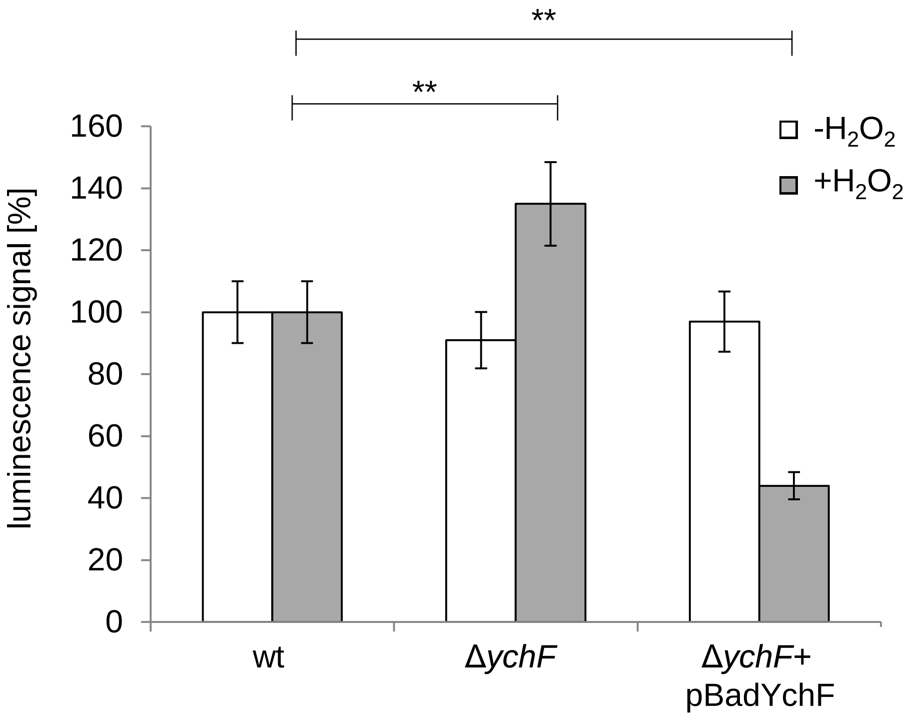

(Landwehr et al, Figure S1)

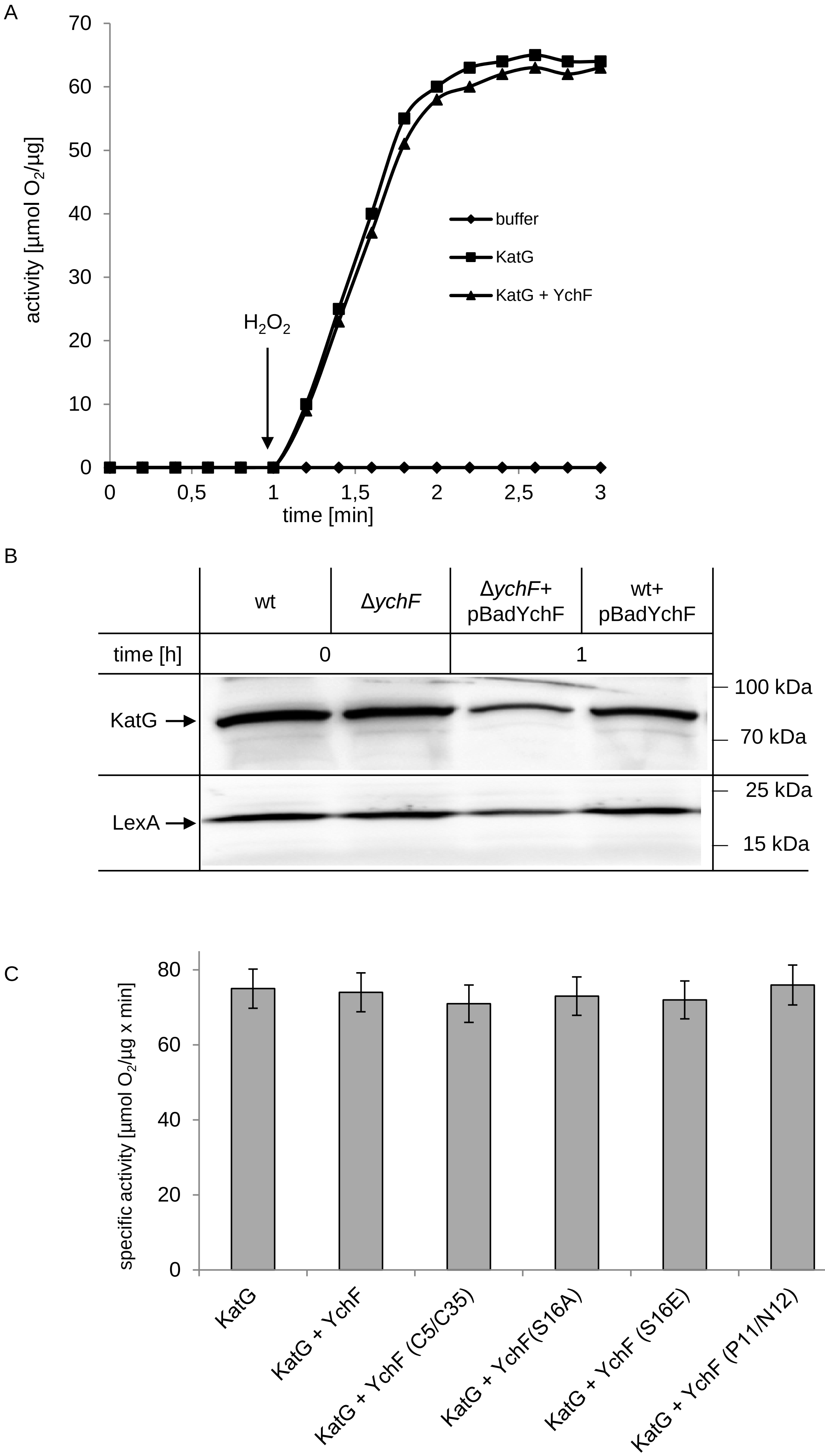

(Landwehr et al, Figure S2)

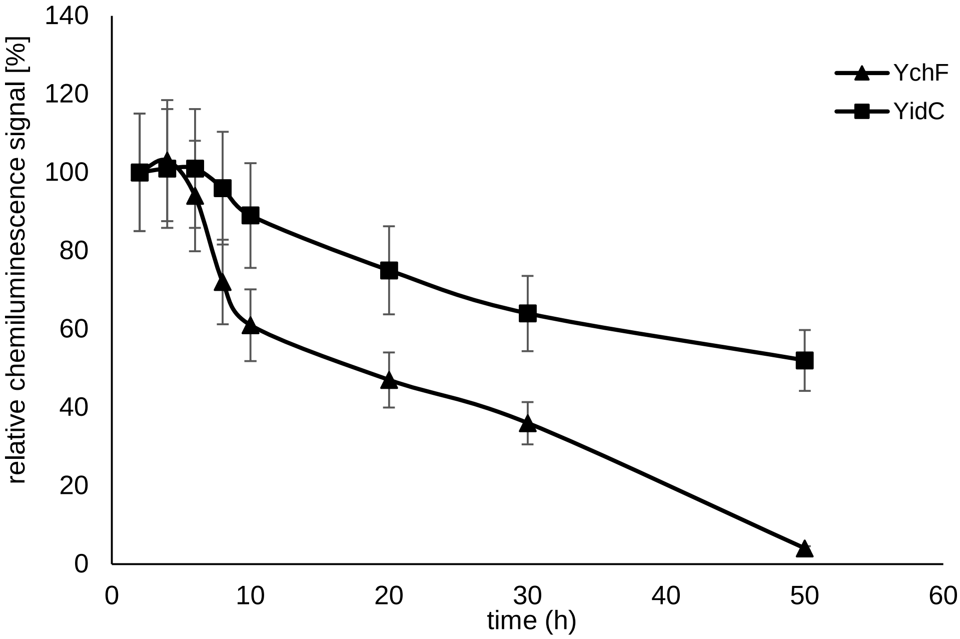

(Landwehr et al, Figure S3)

A

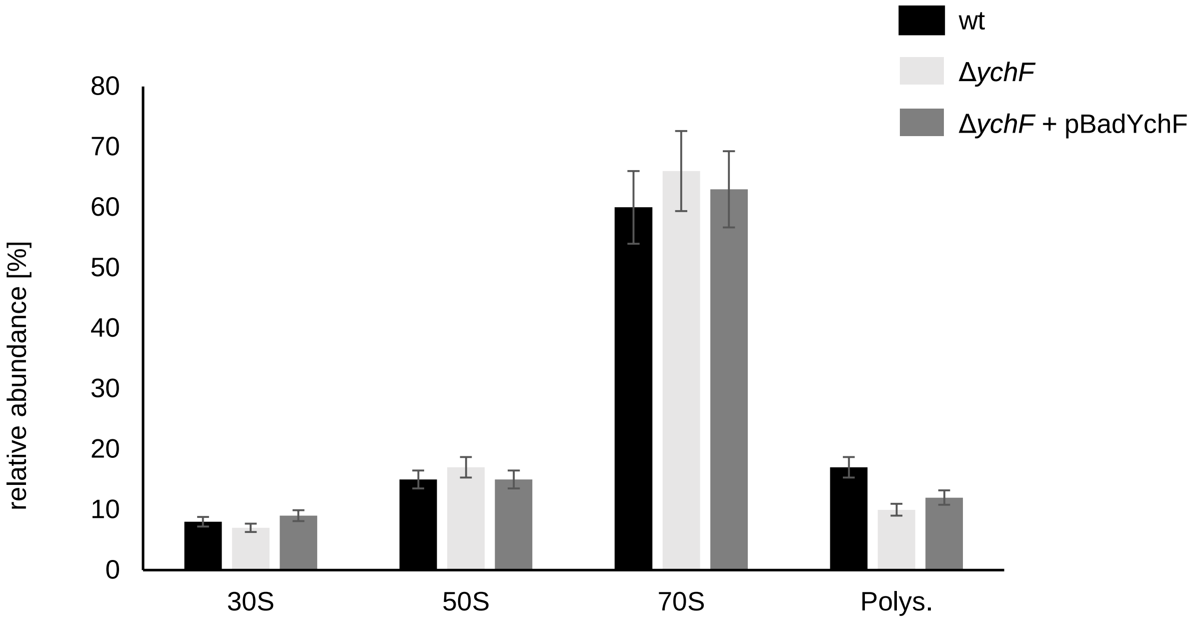

B

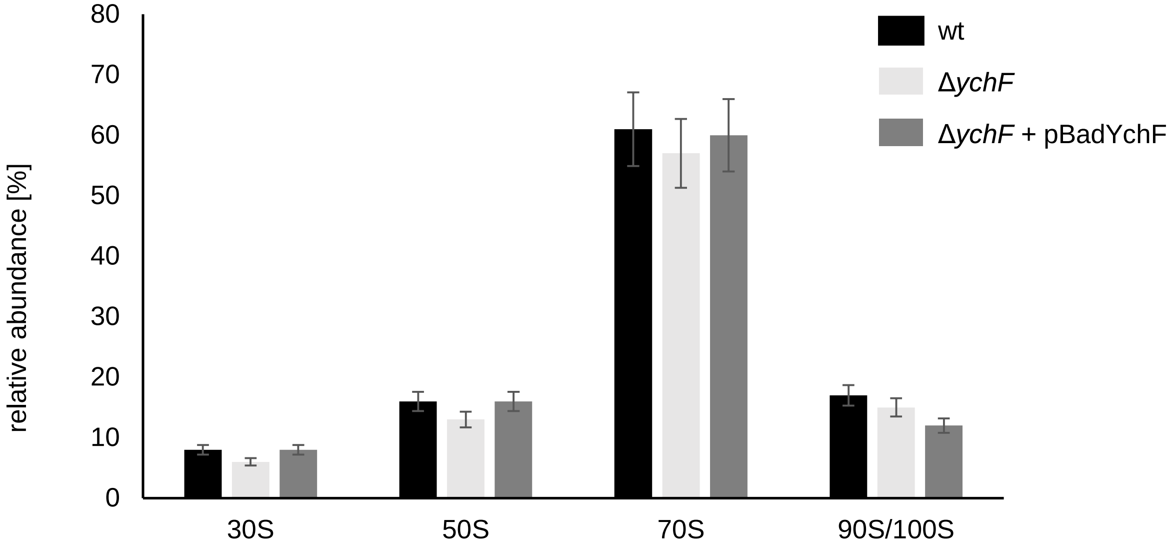

(Landwehr et al, Figure S4)

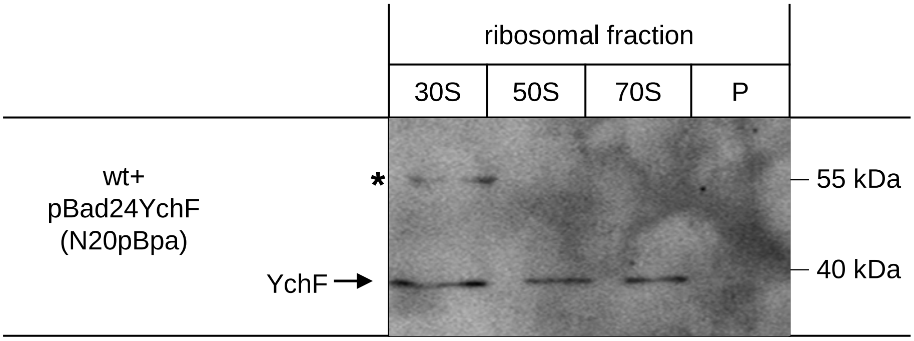

(Landwehr et al, Figure S5)

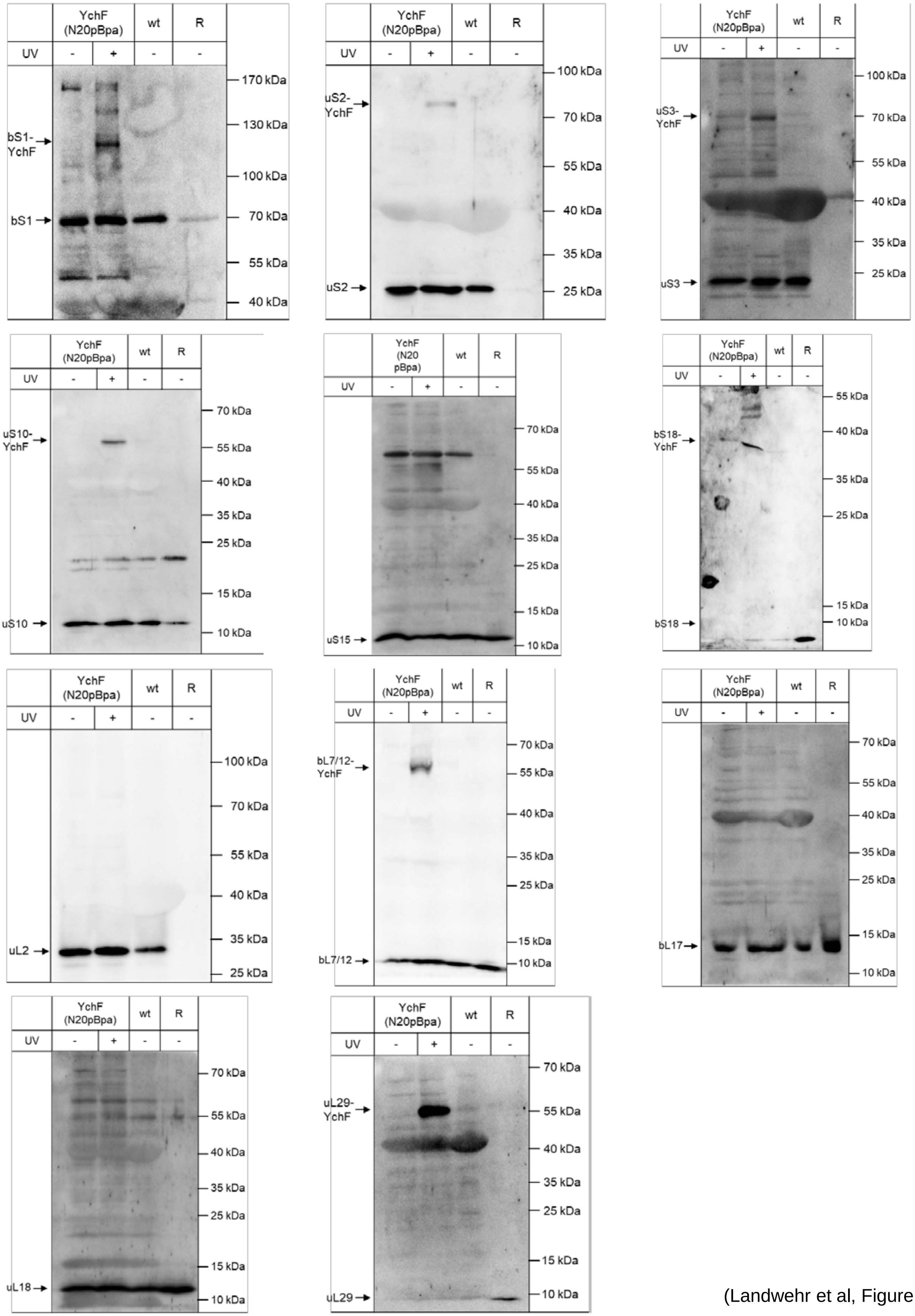

(Landwehr et al, Figure S6)

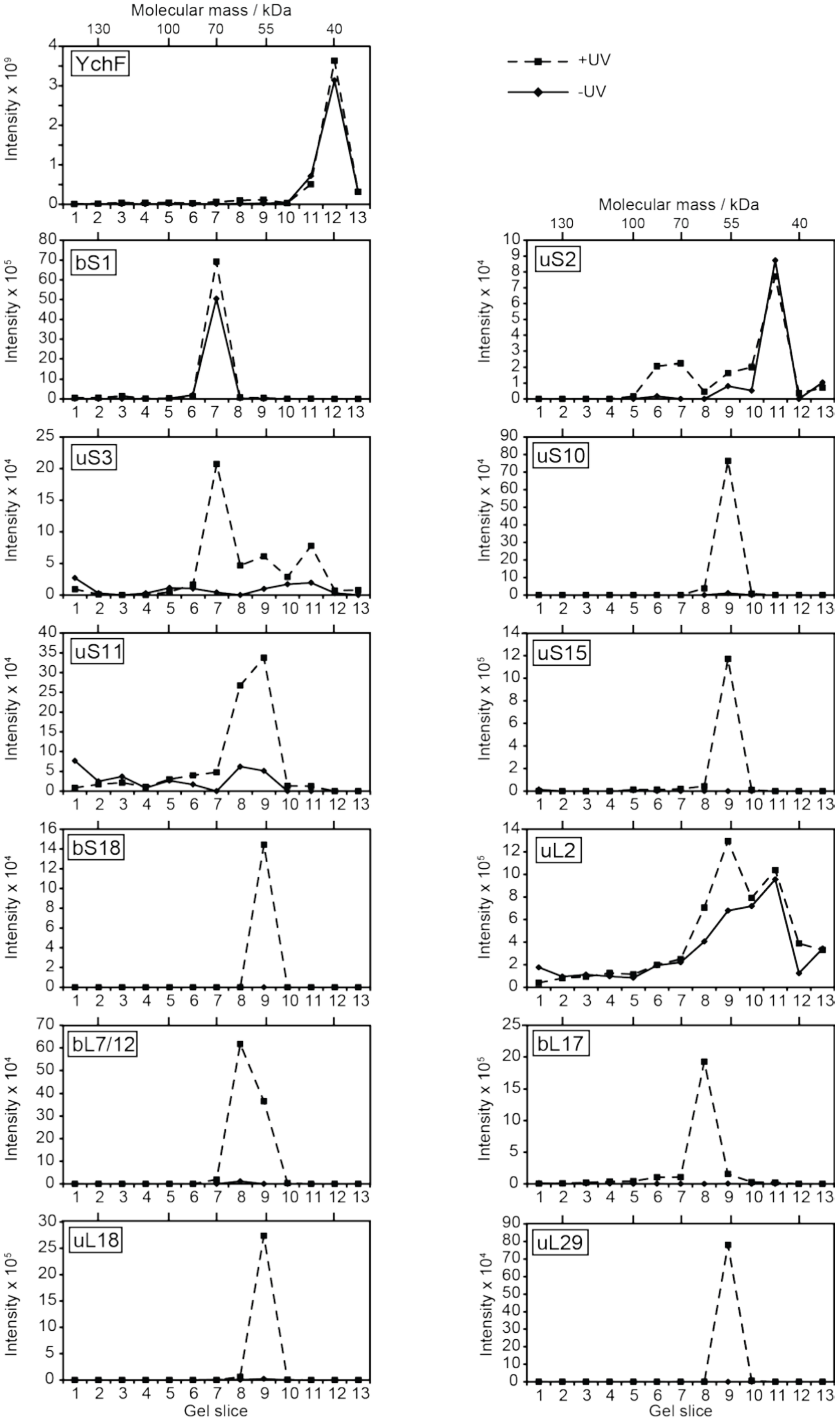

(Landwehr et al, Figure S7)

plasmid	$YchF_{His}$(N20pBpa)		$YchF_{Strep}$(N20pBpa)	
UV	-	+	-	+

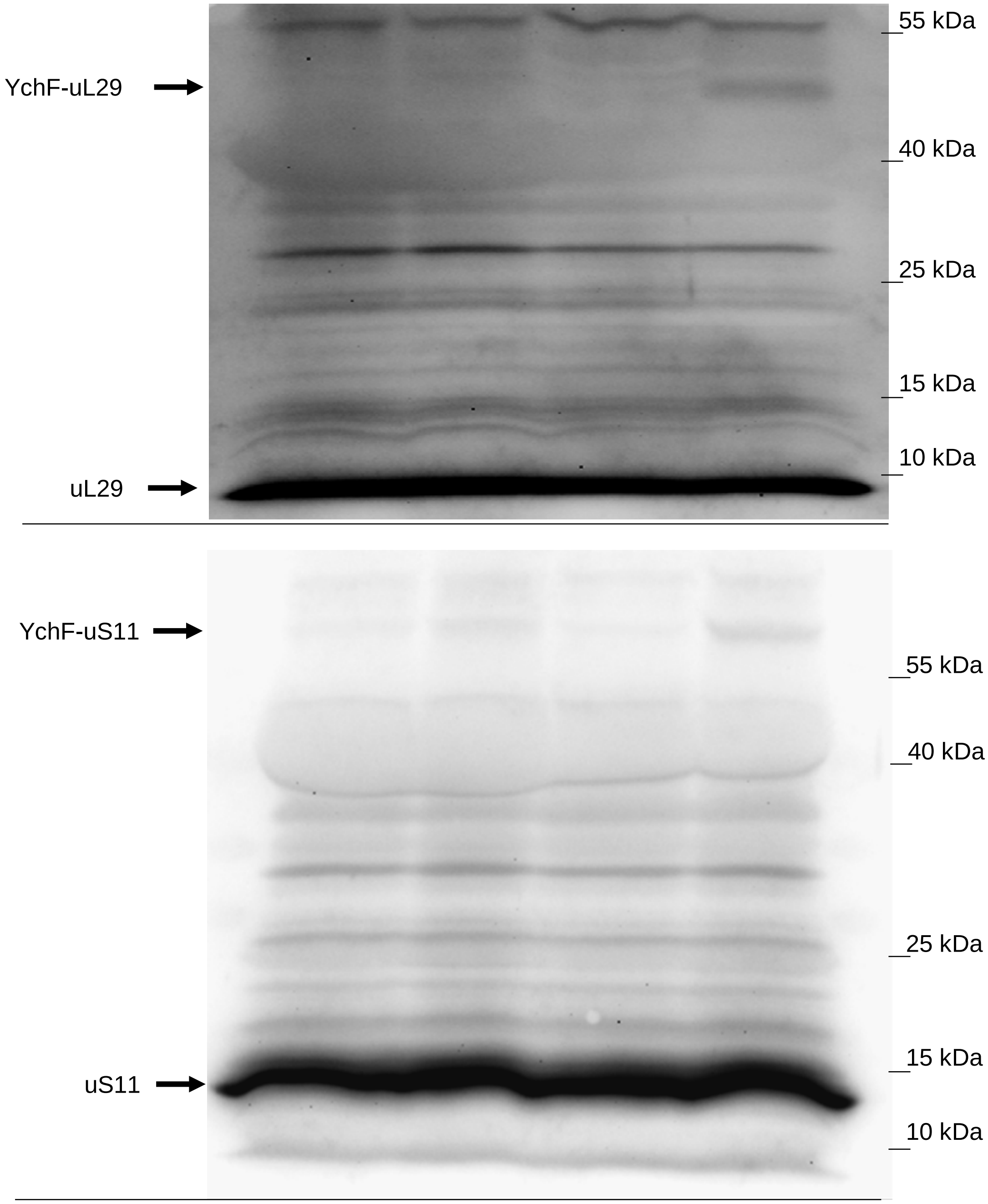

(Landwehr et al, Figure S8)

A

strain	Δ*ychF*		empty	wild type		empty	MW	Δ*ychF* pBadYchF	
plasmid	pCDF-991	pCDF-999		pCDF-991	pCDF-999			pCDF-991	pCDF-999

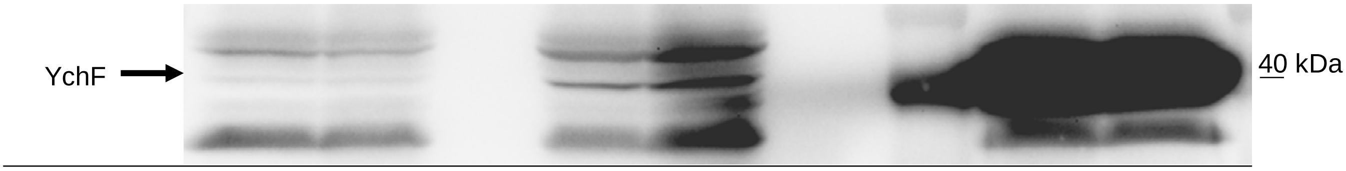

B

strain	wild type		Δ*ychF*		Δ*ychF* pBadYchF	
plasmid	pCDF-991	pCDF-999	pCDF-991	pCDF-999	pCDF-991	pCDF-999

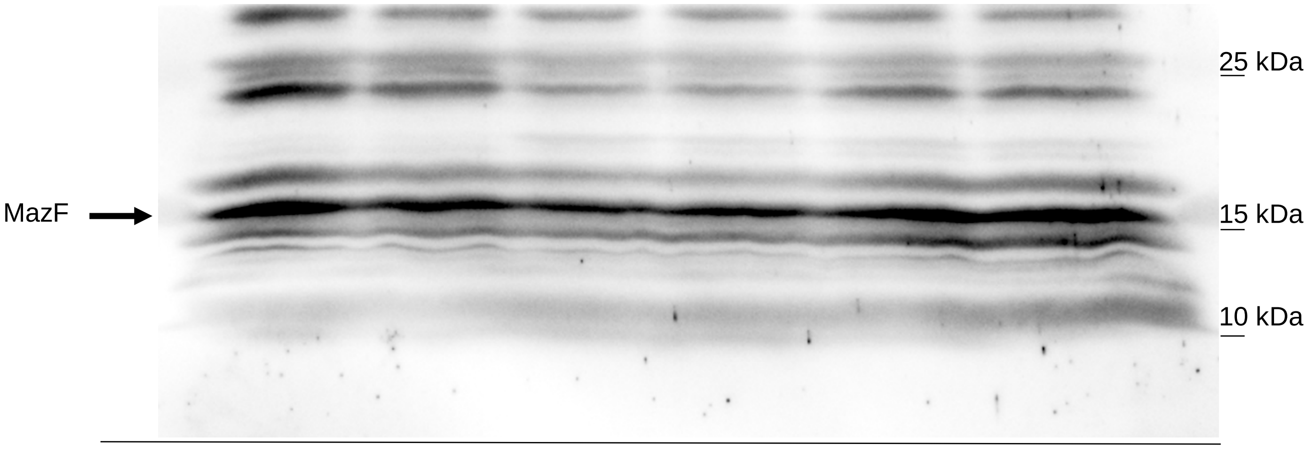

(Landwehr et al, Figure S9)
